# A Comprehensive Overview of TCP Congestion Control in 5G Networks: Research Challenges and Future Perspectives

**DOI:** 10.3390/s21134510

**Published:** 2021-06-30

**Authors:** Josip Lorincz, Zvonimir Klarin, Julije Ožegović

**Affiliations:** 1 Faculty of Electrical Engineering, Mechanical Engineering and Naval Architecture (FESB), University of Split, 21 000 Split, Croatia; julije.ozegovic@fesb.hr; 2 Polytechnic of Sibenik, Trg Andrije Hebranga 11, 22 000 Sibenik, Croatia; zklarin@vus.hr

**Keywords:** TCP, congestion control, 5G, mmWave, network, mobile, algorithms, communications, wireless

## Abstract

In today’s data networks, the main protocol used to ensure reliable communications is the transmission control protocol (TCP). The TCP performance is largely determined by the used congestion control (CC) algorithm. TCP CC algorithms have evolved over the past three decades and a large number of CC algorithm variations have been developed to accommodate various network environments. The fifth-generation (5G) mobile network presents a new challenge for the implementation of the TCP CC mechanism, since networks will operate in environments with huge user device density and vast traffic flows. In contrast to the pre-5G networks that operate in the sub-6 GHz bands, the implementation of TCP CC algorithms in 5G mmWave communications will be further compromised with high variations in channel quality and susceptibility to blockages due to high penetration losses and atmospheric absorptions. These challenges will be particularly present in environments such as sensor networks and Internet of Things (IoT) applications. To alleviate these challenges, this paper provides an overview of the most popular single-flow and multy-flow TCP CC algorithms used in pre-5G networks. The related work on the previous examinations of TCP CC algorithm performance in 5G networks is further presented. A possible implementation of TCP CC algorithms is thoroughly analysed with respect to the specificities of 5G networks, such as the usage of high frequencies in the mmWave spectrum, the frequent horizontal and vertical handovers, the implementation of the 5G core network, the usage of beamforming and data buffering, the exploitation of edge computing, and the constantly transmitted always-on signals. Moreover, the capabilities of machine learning technique implementations for the improvement of TCPs CC performance have been presented last, with a discussion on future research opportunities that can contribute to the improvement of TCP CC implementation in 5G networks. This survey paper can serve as the basis for the development of novel solutions that will ensure the reliable implementation of TCP CC in different usage scenarios of 5G networks.

## 1. Introduction

**Figure 1:**
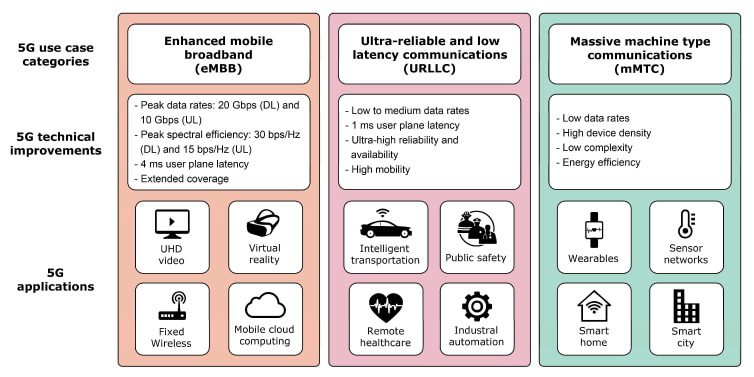
Three main use cases of 5G mobile networks.

For every new generation of mobile network, the demand for new services and use cases increases. Today’s trends of ubiquitous computing tend to incorporate technology into every device, changing the way that we perform our daily activities and do business. Accommodating these trends imposes implementation and operation challenges for mobile network operators. To satisfy future needs, the International Telecommunications Union–Radiocommunications Sector (ITU-R) has defined three main technology advancements that fifth-generation (5G) mobile networks should support (Figure 1) [1]: ultra-reliable and low latency communications (URLLC), enhanced mobile broadband (eMBB), and massive machine-type communications (mMTC) in sensor networks.

The usage scenarios characterised as eMBB aim to provide high data rates, high traffic capacity, and high seamless mobility for both hotspots and wide-area coverage [1]. As 5G mobile technology is implemented in phases, its full potential being reached in the upcoming years will enable eMBB use cases to benefit the majority of the population in this early phase of 5G mobile network development (Figure 1). The reason for this is that the initial implementation of 5G networks is aligned with the consumer market needs, supporting a rising number of smartphone subscriptions accompanied by an increasing mobile data growth rate [2]. In Ref. [3], the minimum requirements for the peak data rate in the eMBB usage scenario are defined as 20 Gbit/s for downlink, 10 Gbit/s for uplink, and 4 ms for user plane latency. These performance targets are assumed for scenarios with a single user transferring small internet protocol (IP) packets for both the downlink and uplink. 

The URLLC use case presents applications that require ultra-low latencies, reliable communication and high availability (Figure 1). High reliability and low latency are crucial requirements for emerging mission-critical applications including telesurgery, intelligent transportation and industrial automation [4]. In URLLC usage scenarios, the minimum requirement for user plane latency for small IP packets is 1 ms for both downlink and uplink transmission [3].

Machine-to-machine communication (M2M) represents machine-centric communication between different devices and sensors without human interaction (Figure 1). With the increasing number of devices and sensors offering M2M communication, mMTC use cases will become more present in the near future. The main driver of increasing machine-type communication is the advent of sensor networks as part of the Internet of Things (IoT) paradigm. This is where a very large number of devices and sensors will communicate and exchange information. These devices and sensors are characterised as low cost and low power with a very long battery life. These devices and sensors are usually transmitting a small amount of data that is not sensitive to delays [1].

Previous generations of cellular networks e.g., 3rd (3G) and 4th (4G), have used frequency bands under 6 GHz. As these frequencies are becoming increasingly saturated and as there are limitations that mean that they cannot fulfil the new increasing demands, the need for higher frequency bands above 6 GHz has arisen. Accordingly, 5G mobile networks are designed to operate on a variety of frequency bands, including the sub-6 GHz band used by the previous generations of mobile networks and the above 6 GHz frequency bands as well. 

This is why 5G technology is also known as 5G New Radio (NR). For the first time in the history of mobile network generations, information exchange uses a frequency spectrum known as millimetre-wave (mmWave) characterized with signal transmission at carrier frequencies larger than 24 GHz. The main reason for using these frequencies is to accommodate the emerging high requirements for the larger data rates [5]. Within the mmWave frequency range, up to 100 GHz of a possible new spectrum is expected to be available for cellular mobile communications [6]. In contrast to the previously available spectrum in the sub-6 GHz band, this represents a significant spectrum increase aimed for usage in 5G networks. 

**Figure 2:**
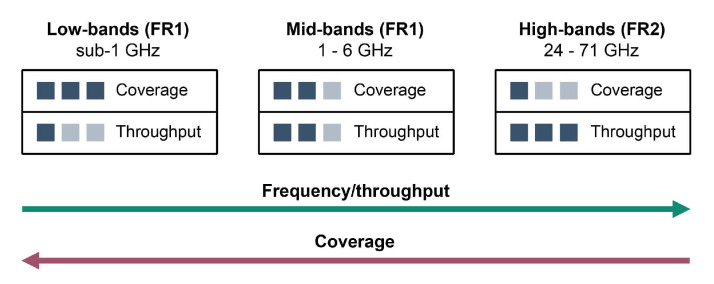
Frequency bands in the 5G networks.

Although mmWave frequency bands classified by the 3rd generation partnership project (3GPP) as FR2 are expected to bring major throughput improvements in mobile communications, sub-6 GHz frequencies classified by 3GPP as FR1 frequency band will still be predominantly used in the initial launches of 5G networks. To accommodate specific purposes, the 5G frequency spectrum is divided into three broad categories (Figure 2): low-bands (sub-1GHz), mid-bands (1–6 GHz), and high-bands (24–52 GHz) [7]. Low-bands are required for wide-area coverage, signal coverage inside buildings and to support IoT use cases. Low-band frequencies of 600–900 MHz are mainly considered across the world [8]. The mid-band spectrum offers a trade-off between coverage and capacity. Most of the commercial 5G networks will use 3.3 GHz to 4.2 GHz range in the mid-band spectrum [8]. High-band frequencies are required to achieve ultra-high data rates and ultra-low latencies that are expected in 5G communication. Allocated global mmWave spectrum includes 24–28 GHz frequencies, with more spectrum allocation planned in the future (e.g., 37–50 GHz, 50–71 GHz) [8].

The main advantage of deploying mmWave frequencies is the ability to deliver high data rates using the large bandwidth. Due to the short wavelengths (below 10 mm) of the high-frequency waves, transmitting and receiving antennas can be produced in compact sizes and easily implemented within base stations (BSs) and user equipment (UE). Ultra-dense antenna arrays deployed within a BS can produce narrow directional beams, and therefore enable frequency reuse among concurrent transmissions within a small geographic area [9]. 

However, the propagation attributes of mmWave frequencies have some drawbacks that 5G mobile networks need to overcome. The main drawbacks that are facing mmWave communication systems are increased path loss, susceptibility to blockages and atmospheric absorption [10]. The use of high frequencies implies that the transmitted signals have short wavelengths, which are very sensitive to the blockages caused by various obstacles [11]. Additionally, user mobility causes shifts in the mmWave communication from both line-of-sight (LOS) and non-line-of-sight (NLOS) transmissions. This results in severe signal degradation due to the various objects blocking the signal propagation. 

Some approaches such as massive multiple-input and multiple-output (mMIMO) transmissions with beam-forming and small cell densification have been developed to address these shortcomings. However, a problem that arises using directional beamforming is beam misalignment. This problem is characterised with transmitter’s beam not aligned with the receiver’s beam, causing signal degradation or even a loss of connection.

Besides affecting the physical and media access control (MAC) layers, ensuring the high data rates in environments characterised with fluctuations in channel quality will also deteriorate the performance of the transport layer of the network [12]. Ensuring reliable end-to-end connections over 5G mobile networks, especially when operating in the mmWave spectrum, presents a demanding challenge in terms of its practical implementation. This challenge arose during the last years of the subject of the researchers’ interest [13]. In the transport layer, the transmission control protocol (TCP) is a major protocol used in today’s communication over the Internet. The TCP is designed to provide reliable end-to-end connection over unreliable links. One of the mechanisms that plays a critical role in TCP performance is congestion control (CC). 

One of the main purposes of the CC mechanism is to probe the network for available capacity by exponentially increasing the window size. This approach enables avoiding the congestion of the network that can be caused by a large burst of data at the beginning of transmission [14]. Blockage and misalignment problems can seriously affect the TCP CC mechanism, resulting in poor end-to-end transmission performance. Conventional TCP CC algorithms are not able to differentiate between the potential causes of packet loss. They can occur due to congestion or poor channel quality caused by the blockage or beam misalignment [15]. To achieve the specified performance and to accommodate the 5G requirements, the aforementioned shortcomings need to be addressed. In this paper, the TCP CC performance in 5G mmWave communication systems has been analysed. The paper investigates the recent works regarding TCP CC in 5G networks and discusses the opportunities and future research challenges related to improving TPC CC in 5G networks operating in the sub-6 GHz and mmWave spectrum. 

The rest of the paper is organised as follows. The TCP CC mechanism is explained and a brief review of the popular single and multipath CC algorithms is presented in Section 2. In Section 3, the investigation into the related works dedicated to the CC algorithms used in 5G networks is given. Section 4 overviews the future challenges in the realisation of TCP CC regarding the different technological advancements that will be implemented in 5G networks. In Section 5, an overview of the possible implementation of machine learning for improving TCP CC in 5G networks is presented. The research challenges and future directions that can contribute to enhancing TCP CC in 5G networks are discussed in Section 6. Finally, some concluding remarks are given in Section 7. 

## 2. TCP Congestion Control

The TCP is a connection-oriented, reliable end-to-end protocol that guarantees the ordered delivery of byte streams in a full-duplex inter-process communication [16,17]. Initially, TCP was developed as a protocol to support military computer communication systems. Nowadays, TCP is de facto the standard for reliable end-to-end communication over the Internet. The TCP is responsible for establishing and terminating the connection, the reliable communication between hosts, the flow control, and the CC over unreliable networks. 

As defined in Ref. [17], the process of establishing a TCP connection involves a three-way handshake (3WHS) procedure. Since the 3WHS process increases the latency of TCP flows, especially short flows that are common in today’s web services, a new mechanism called TCP fast open (TFO) has been proposed [18]. TFO is an optional mechanism that decreases latency by eliminating one full round-trip-time (RTT). This RTT elimination is performed by allowing data exchange before the standard 3WHS process is completed. This is achieved by enabling data packets to be transferred in SYN and SYN-ACK packets, and as such delivered to the application at receiving end [18]. TCP uses a flow control mechanism to determine how much data the receiver is able to accept. The receiver uses a window size field in the TCP header, which represents the available buffer size of the receiver. This informs the sender about the maximum number of bytes that they are allowed to transmit [19]. The purpose of the TCP CC mechanism is to avoid congestion of the network.

### 2.1. Network Congestion and TCP CC Mechanism

Network congestion is the result of a network node being overwhelmed with more data than it can process. Buffers are added to the communication nodes to prevent packet loss caused by the burst of packets. This consequently increases the delay of each packet passing through the buffer, which results in the degradation of the network performance [20]. On the other hand, network nodes with large buffers can cause an excess buffering of the packets. This is known as a bufferbloat problem [21]. This consequently leads to long queuing delays and overall network throughput degradation. The maximum bandwidth of the network link is referred to as a bottleneck bandwidth and it is determined by the bandwidth of the fully saturated network link. In a scenario where the TCP data rate is less than the bottleneck bandwidth, no congestion occurs and the delivery rate corresponds to the sending rate [20]. As the sending rate exceeds the bottleneck bandwidth, the buffers start to fill to the point where the buffers are full, causing the network nodes to start dropping packets. The optimal operation point of TCP is the point where the sending rate is equal to the bottleneck bandwidth. The TCP CC is a mechanism that aims to prevent network congestion and to ensure efficient network utilisation while working near to the optimal operation point. 

The TCP CC is one of the main parts of the TCP protocol. Over the years, it has experienced numerous improvements through the application of different algorithms. The first TCP specification considered only flow control as a mechanism to prevent the buffer overflowing on the receiver end, while neglecting the congestion of the network itself [17]. The general idea of TCP CC is to prevent the sender from overflowing the network by determining the available capacity. Congestion avoidance and control were first introduced in 1988 [22] after a series of “congestion collapses” occurred on the Internet. The standard TCP CC mechanism is based on the additive increase multiplicative decrease (AIMD) algorithm, which incorporates four phases: slow start, congestion avoidance, fast retransmit and fast recovery [14]. An initial version of CC only requires the implementation of a slow start and the congestion avoidance phase. The fast retransmit and fast recovery phases were introduced later. 

#### 2.1.1. The Slow Start and Congestion Avoidance TCP Mechanism

**Figure 3:**
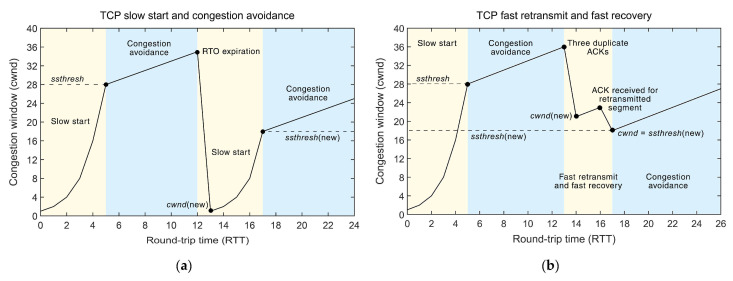
Standard working principles of the TCP CC mechanism related to the interdependence between *cwnd* and RTT for: (**a**) the TCP slow start and congestion avoidance mechanism; (**b**) the TCP fast retransmit and fast recovery mechanism.

The interdependence between the congestion window (*cwnd*) size and the transmission RTT of the TCP segment for the TCP version based on a slow start with congestion avoidance has been presented in Figure 3a. In this approach, the slow start and congestion avoidance phases are implemented using a stated *cwnd* variable that controls the amount of data that can be sent before receiving an ACK (Figure 3a). The *cwnd* size is a sender-specific variable that is maintained for each TCP session. The bandwidth-delay product (BDP), which determines the maximum amount of incoming data over a network link is considered to be the ideal value of the *cwnd* size. The network congestion is evaluated based on the received ACK packets of transmitted data. The slow start phase is the initial phase in the beginning of transmission (Figure 3a). In this phase, the TCP quickly probes the available capacity while avoiding the network congestion that can occur in the beginning of the transmission. After every successful transmission of data and the reception of an appropriate ACK for said data, *cwnd* is increased and the sender can send more data (Figure 3a). After the three-way handshake process, the initial window (*iw*) is determined and can be large as 10 maximum segment size (MSS) [23]. In the slow start phase, the *cwnd* is increased by one MSS for each ACK received, doubling its size for every RTT. The *cwnd* will continue increasing the rate until either the *ssthresh* (slow-start threshold) is reached (Figure 3a), packet loss occurs or *cwnd* exceeds the *rwnd* (receiver window) size.

After *cwnd* exceeds the *ssthresh* size, the TCP CC mechanism enters the congestion avoidance phase (Figure 3a). The purpose of the congestion avoidance phase is to slowly probe the network for more available capacity, by increasing the *cwnd* less aggressively than in the slow start phase. The connection stays in the congestion avoidance phase until congestion is detected. If congestion is detected during the congestion avoidance phase using the retransmission time-out (RTO) expiration parameter, the TCP connection enters the slow start phase where the *ssthresh* is set to half of the current *cwnd* size and the *cwnd* is decreased to one MSS (Figure 3a). The process of adjusting the congestion window (*cwnd)* continues according to the described mechanism. 

#### 2.1.2. The Fast Retransmit and Fast Recovery TCP Mechanism

Waiting for the RTO to expire leads to a long period of connection inactivity. Because of that, a new mechanism called fast retransmit with fast recovery was introduced as a part of the TCP CC [24] (Figure 3b). The interdependence between the congestion window (*cwnd*) size and the transmission RTT of the TCP segment for the TCP version based on a fast retransmit with a fast recovery mechanism has been presented in Figure 3b. The general idea of a fast retransmit with a fast recovery is not to replace the RTO parameter, but to work in parallel, allowing the sender to retransmit the lost segment even if the timeout has not expired. In a TCP communication, when a segment arrives out of order, the receiver resends the ACK that was last sent, causing the sender to receive duplicate ACKs. This signals to the sender that the segment is either lost or delayed. This causes a reordering of the segments. In the case of segment reordering, it is assumed that only one or two duplicate ACKs will be sent. The case when three ACKs are received by the sender before the RTO has expired indicates that the segment is lost. This triggers a fast retransmit and a fast recovery mechanism (Figure 3b). In the standard implementation of the fast retransmit with a fast recovery phase, the sender, instead of going into the slow-start phase, decreases the *ssthresh* by one-half of the current *cwnd* (but by no less than two segments). Additionally, the sender retransmits the missing segment and sets the *cwnd* to a value equal to *ssthresh* plus 3 times the segment size [24] (Figure 3b). Each time, the sender receives an additional duplicate ACK after the third *cwnd* is incremented by MSS. When the ACK that acknowledges the new data arrives, *cwnd* is set to the new value of *ssthresh* (which is set at the moment of the initial triggering of the mechanism). It then enters the congestion avoidance phase, as shown in Figure 3b [24]. 

The aforementioned TCP CC mechanism is the standard implementation of the mechanism. It can vary depending on the CC algorithm used. There are numerous CC algorithms that have been developed and proposed as CC emerged as one of the most studied areas of internet research during the last few decades [25]. TCP CC, although not part of the original TCP implementation, has become an essential element of TCP as the entire TCP performance depends on the CC algorithm. 

### 2.2. TCP CC Algorithms 

**Figure 4:**
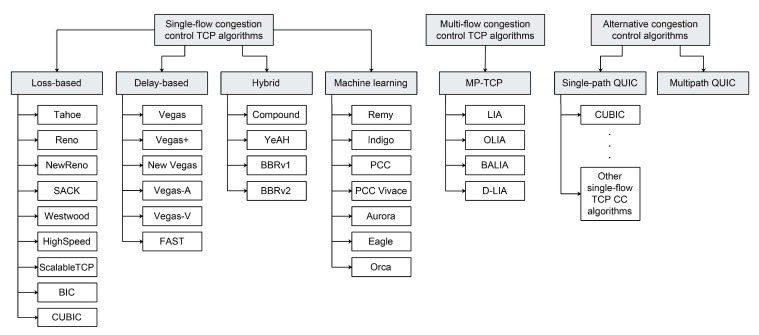
Main categories and types of the TCP CC algorithms.

CC algorithms are being developed with a focus on optimizing different metrics and making trade-offs between the various metrics in order to accommodate the different working environments and use cases. The main metrics that the Transport Modelling Research Group (TMRG) of the Internet Research Task Force proposed for evaluating CC algorithms were [26]: throughput, delay, packet loss rates, fairness, convergence times and robustness. Depending on the desired working environment, TCP CC algorithms are usually designed to make trade-offs between these metrics. There are three main categories of TCP CC algorithms as shown in Figure 4: (1) loss-based algorithms that use packet loss as an indicator of congestion, (2) delay-based algorithms that predict packet loss based on RTT measurements and (3) hybrid algorithms in which, the loss-based and delay-based methods are combined. Table 1 presents the comparison of the different CC algorithms according to their classification and year of introduction.

#### 2.2.1. Loss-Based TCP CC Algorithms

A Tahoe TCP was the first CC algorithm that implemented a fast retransmit phase [22], followed by the Reno TCP [27] which included a fast recovery procedure (Table 1). The Tahoe TCP and Reno TCP are loss-based algorithms, and both consider RTO and duplicate ACKs as an indication of segment loss due to network congestion. The main difference between the two CC algorithms is in how they respond after receiving three duplicate ACKs and how they perform the fast retransmitting of the segments. After the fast retransmit phase, the Tahoe TCP switches to the slow start phase, whereas in contrast, the Reno TCP avoids the slow start phase by entering the fast recovery phase. In high-traffic environments where multiple segment losses occur in a single congestion window, the Reno TCP has shown a performance decrease as it can only detect single-segment losses [28]. 

TCP NewReno is an improved version of the TCP Reno algorithm. In order to overcome the performance issues of the TCP Reno, the TCP NewReno introduces a slight modification of the TCP Reno’s fast recovery mechanism (Table 1) [29]. After receiving multiple duplicate packets, like its predecessor, the TCP NewReno enters the fast retransmit phase. However, the TCP NewReno does not exit the fast recovery phase until all of the outstanding data (send without received ACK) at the time of entering the fast recovery phase is acknowledged, thus preventing multiple *cwnd* reductions.

TCP selective acknowledgments (SACKs) have been introduced to overcome the limitations of the algorithms that use cumulative ACKs (Table 1). This approach allows for the retransmission of more than one segment per RTT. In an established TCP connection, the receiver uses the selective ACKs (SACKs) option to inform the sender about all successfully received segments, thus allowing the sender to retransmit only the missing segments in one RTT [30]. 

TCP Westwood is a CC algorithm that uses a server-side modification of the TCP Reno *cwnd* control mechanism (Table 1) [31]. TCP Westwood improves the performance of TCP Reno, especially in lossy wireless networks due to its robustness against sporadic wireless network errors. It uses a mechanism called faster recovery where instead of halving *cwnd* after three duplicate ACKs, the mechanism adjusts the *cwnd* and *ssthresh* parameters based on the end-to-end estimation of the available bandwidth. The bandwidth of the connection is estimated through the regular monitoring of the ACKs returning rate. After the congestion event, the size of the *cwnd* and *ssthresh* parameters is set according to the bandwidth estimate, thus ensuring a faster recovery as opposed to the TCP Reno response [31].

HighSpeed TCP is a modification of the classical TCP CC mechanism [14] that aims to address the limitations of connections with large congestion windows (Table 1) [32]. In the situation of a small *cwnd*, the operation of the HighSpeed TCP is the same as in the standard CC. On the other hand, when the current *cwnd* is greater than the specified parameter, the HighSpeed TCP uses a modified TCP response function that allows for a faster *cwnd* increase rate and a faster recovery time after packet loss. A variation of the HighSpeed TCP known as the Scalable TCP [33] is optimised for high-speed wide area networks. It is based on a simple modification of the traditional CC mechanism [14]. The main goal of the Scalable TCP algorithm is to update the *cwnd* in a scalable fashion. This means that the recovery times are proportional only to the RTT of the connection, which makes the algorithm more robust in high BDP networks.

A binary increase congestion control (BIC) TCP algorithm has been developed to address unfairness issues and to provide bandwidth scalability in high-speed networks with large delays (Table 1) [34]. The BIC TCP algorithm uses a binary search increase and additive increase as the two techniques used to determine the *cwnd* size. Binary search increase is a search technique involving aggressive initial bandwidth probing in a situation where there is a large difference between the current *cwnd* size and the target *cwnd* size. The mechanism becomes less aggressive when the current *cwnd* size gets closer to the target *cwnd* size. In the case of network congestion, the BIC TCP reduces *cwnd* from performing multiplicative decreases. After a significant *cwnd* decrease, the BIC algorithm performs an additive increase scheme by increasing *cwnd* linearly. The increase then becomes logarithmic [34]. By combining the binary search increase with the additive increase technique, the BIC algorithm ensures RTT fairness and faster convergence times.

**Figure 5:**
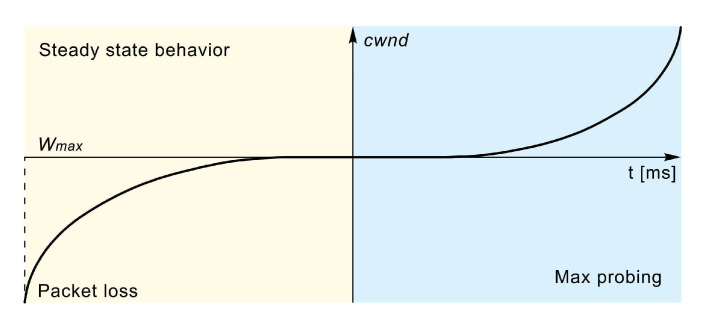
TCP CUBIC window growth function after the packet loss event.

The authors of [35] proposed a high-speed TCP variant called CUBIC to overcome the fairness and complexity issues of the previous CC algorithms (such as the TCP BIC). TCP CUBIC has become the dominant CC algorithm on the Internet [46] and it is currently the default algorithm distributed by Linux (Table 1). The performance of the TCP CUBIC algorithm is visualised in Figure 5. To adjust the *cwnd* size, the TCP CUBIC uses a cubic function that is characterised by the aggressive growth of the window size following the event when a packet loss is detected. When the *cwnd* size approaches the point of the last congestion event (marked as $W_{max}$ in Figure 5), it slows its growth to almost zero. After reaching the $W_{max}$ point, the algorithm slowly probes the network by increasing the *cwnd* size at a slow rate. As it moves away from the $W_{max}$, it rapidly accelerates its growth rate (Figure 5). Using a cubic function for calculating the *cwnd* growth rate ensures fairness among the concurrent flows and good performance properties [25].

#### 2.2.2. Delay-Based TCP CC Algorithms

All aforementioned loss-based CC algorithms use segment loss as a congestion indicator and they are based on utilising a reactive congestion adaptation method. These algorithms are opposed to proactive delay-based CC algorithms (Figure 1) [25]. A typical representative of loss-based CC algorithms is TCP Vegas (Figure 4). This was introduced as a delay-based modification of the TCP Reno algorithm (Table 1). It predicts network congestion before actual segment loss occurs. TCP Vegas uses a fine-grained RTT estimation that increases the accuracy of the computed timeout period, which leads to a very efficient segment retransmission schedule (Table 1) [36]. The TCP Vegas uses a modified retransmission mechanism based on calculating the RTT estimation used for detecting segment loss before waiting for the reception of the three duplicate ACKs (Table 1). This approach increases the algorithm recovery time. It also uses a modified version of the slow start and congestion avoidance phases, which are thoroughly explained in Ref. [36]. The TCP Vegas delay-based approach can improve the overall TCP flow throughput by keeping the sending rate stable. However, due to the slow *cwnd* growth, this approach can suffer from performance issues in high-speed networks. In networks with concurrent loss-based flows, the TCP Vegas algorithm shows a decrease in performance due to the unfair resource allocation [25]. 

In the literature, several modifications of the TCP Vegas algorithm have been proposed. They are dedicated to addressing the limitations of bottlenecks during link sharing when many TCP flows are present, and to alleviate the performance issues in the case of links with high latency. A modified version of the TCP Vegas, called the TCP Vegas+ has been introduced to overcome fairness issues between the TCP Reno and the TCP Vegas (Figure 4) [37]. The algorithm introduced an aggressive mode to maintain fair throughput when competing with TCP Reno flows. To address performance issues of TCP Vegas over high latency links, a TCP New Vegas (Figure 4) has also been introduced. Compared with TCP Vegas, TCP New Vegas implements three sender-side modifications of TCP Vegas [38]. Furthermore, another modification of TCP Vegas has been developed, called TCP Vegas-A (Figure 4). The algorithm uses the modified congestion avoidance mechanism of TCP Vegas to address issues such as fairness against TCP New Reno flows, rerouting issues, bias against high bandwidth connections and fairness issues between old and new connections in wired and satellite networks [39]. The authors in Ref. [40] proposed a modified version of TCP Vegas-A, called TCP Vegas-V (Figure 4). This CC algorithm improves performance regarding fairness and aggression features in the network environment containing competing TCP Vegas, TCP Vegas-A and TCP Reno flows. 

Another representative of delay-based CC algorithms is the FAST TCP algorithm that is designed for high-speed long-latency networks (Table 1) [41]. Based on the feedback information of the average RTT and average queuing delay, the FAST TCP periodically updates *cwnd* in order to control the packet transmission. This feedback information is provided by a latency estimation of each packet sent. Fairness and the number of buffered packets in the network are both controlled by a scaling parameter. FAST TCP adjusts its *cwnd* size constantly according to the number of buffered packets. If the number of buffered packets is far from the defined scaling parameter, the FAST TCP algorithm increases or decreases the *cwnd* size, effectively utilising the network capacity. On the other hand, when the number of buffered packets reaches the target scaling parameter, the FAST TCP algorithm adjusts its *cwnd* by a small amount, thus ensuring the networks’ stability [47].

#### 2.2.3. Hybrid TCP CC Algorithms

Hybrid TCP CC algorithms are a combination of loss-based and delay-based algorithms (Figure 4). In a congested network situation or a network with high link utilisation having short bottleneck queues, hybrid algorithms tend to use a delay-based CC approach. On the other hand, in a high-speed network with a low link utilisation, hybrid algorithms tend to use a more aggressive CC approach that is characteristic of loss-based algorithms [20]. Some of the well-known representatives of hybrid CC algorithms are Compound TCP, Yet Another Highspeed (YeAH) TCP, and the TCP Bottleneck Bandwidth and Round-trip Propagation Time (BBR) algorithm (Figure 4). 

The Compound TCP algorithm is representative of a hybrid-based CC algorithm developed for high-speed and long-distance networks. It was the default TCP algorithm in Microsoft Windows operating systems (Table 1) [42]. Compound TCP is designed to ensure high network utilisation, RTT and TCP fairness using a combination of loss-based and delay-based approaches. In the slow start phase, Compound TCP has the same aggressive behaviour characteristic as standard loss-based CC algorithms. During the congestion avoidance phase, it uses a combination of a standard loss-based component and a new scalable delay-based component. The delay-based component is based on the TCP Vegas algorithm, and it is controlled by a new state variable called *dwnd* (delay window). By encompassing the delay-based component, Compound TCP provides faster network utilisation and a reduction of the sending rate according to the bottleneck queue. This ensures better TCP fairness and the reduction of the sending rate in a packet loss event.

YeAH TCP is a hybrid variant of the high-speed TCP CC algorithm which uses two operation modes known as fast and slow modes respectively (Figure 4) [43]. During the fast recovery phase, the fast mode is initiated and the *cwnd* is incremented aggressively as in the Scalable TCP algorithm (Table 1). In the congestion avoidance phase, the slow mode is triggered by adjusting the *cwnd* size in the TCP Reno fashion. YeAH TCP uses the number of packets in the bottleneck queue determined through RTT measurements. This packet number is used as a parameter to determine the state of the algorithm. The main benefits of YeAH TCP are its high efficiency in situations where there are small link buffers typical of high BDP networks. An important benefit of the YeAH TCP algorithm compared to loss-based algorithms is its improved fairness.

The TCP BBR algorithm was developed by Google to overcome the problems faced by loss-based CC algorithms such as throughput issues in a small buffer scenario and the bufferbloat problem in a network with large bottleneck buffers (Table 1) [44]. It is a cutting-edge CC algorithm that creates an explicit CC model based on the recent measurements of the RTT and the delivery rate of the network. Using an explicit CC data model, the algorithm adjusts the sending rate accordingly. According to the periodic estimation of the available bandwidth and minimal RTT, the BBR algorithm prevents congestion by ensuring low delay and high throughput operations. The sending rate is determined through the pacing gain process, which is the main mechanism that the BBR uses to control the sending behaviour. This is done by maximising the rate at which the BBR schedules packets. When the bottleneck bandwidth (*BtlBw*) has been estimated, the BBR deliberately reduces the pacing gain to drain the queues in order to estimate the round trip propagation time (*RTprop*). Periodical measurements of BDP calculated as the multiplication of the *BtlBw* and *RTprop* parameters ensure performance within the Kleinrock optimal operating point [48], where data delivery rate is maximised and delay is minimised [49]. However, the initial version of the BBR has drawbacks which are mainly reflected in unfairness when competing with loss-based CC, including a large number of packet losses and increased queuing delays [50]. For that reason, an improved version known as a BBRv2 has been introduced to alleviate these problems [45]. Google has deployed TCP BBR across all of its services, which has resulted in a higher throughput, reduced latency and better overall connection quality. Even though TCP BBR is a relatively new CC algorithm, [46] showed that after TCP CUBIC, it is the second most dominant CC algorithm used on the Internet. It is reasonable to predict that TCP BBR will surpass TCP CUBIC in the near future.

### 2.3. TCP Fairness

In a situation of multiple concurrent TCP flows competing for the same bandwidth on the network link, some TCP CC algorithms may receive more of a bandwidth share than other TCP flows. The challenge of satisfying bandwidth allocation fairness is a serious problem in TCP CC. Hence, one of the main goals that CC algorithms need to accomplish is to ensure fairness among flows that are competing for the same bottleneck bandwidth [51]. Due to the competition among different TCP flows, fair bandwidth allocation among TCP flows can severely degrade the performance of the CC algorithms characterised by a less aggressive approach in the competition for bandwidth. 

Loss-based CC algorithms (e.g., TCP Reno) have an aggressive nature as packet loss is the only indicator of congestion. This nature is mainly reflected in the continuous increase of *cwnd* until packet loss occurs. On the other hand, delay-based algorithms (e.g., TCP Vegas) use RTT estimation to predict network congestion before an actual loss has occurred, reducing the sending rate accordingly. In a network with many competing flows that are loss-based, delay-based flows cannot get their fair throughput share as shown in Ref. [52]. This is because flows with an aggressive loss-based approach will obtain a larger amount of bandwidth than their fair share. Likewise, the fairness issue has been detected between the most dominant CC algorithms on the Internet, and the CUBIC and BBR algorithms are no exception. It has been shown that TCP BBR favours small buffers and gets a significantly higher share when competing with CUBIC. In contrast, when using large buffers, BBR cannot compete with CUBIC because it occupies most of the bottleneck bandwidth as shown in Ref. [50]. When deploying CC algorithms in dynamic environments, such as the mmWave 5G network where a large number of lines of LOS-NLOS transitions are expected, fairness issues should be seriously considered. The NLOS state of the TCP flow causes an increase in RTT, which consequently results in bandwidth unfairness among the flows [53].

### 2.4. TCP Optimisation Techniques

The purpose of TCP CC mechanisms is to address congestion on the network and to fully utilise the network resources. Besides CC, several TCP optimisation techniques have been developed to enhance the operations of CC. To solve the excessive buffering of packets on the network and thus preventing bufferbloat, active queue management (AQM) techniques are proposed as opposed to a basic drop-tail queuing mechanism. AQM is a technique based on proactively discarding packets from the buffer before the queue is full, thus reducing the risk of a queuing delay, preventing the bufferbloat problem and proactively reducing network congestion [54]. AQM schemes are deployed on the network device buffers (e.g., routers, BSs, etc.), rather than as part of a TCP implementation. The benefit of using the AQM is to maintain a small queue size to prevent overflowing the buffers with a large burst of packets. Furthermore, keeping the queue size small reduces the queuing delay and minimises the overall end-to-end delay of the network. Finally, the AQM avoids the impact of global TCP synchronisation and this contributes to the increased throughput and better utilisation of the network [55]. 

Random early detection (RED) is an example of a basic AQM algorithm that addresses global synchronization problem, minimise network congestion and limits network delay [56]. Due to the configuration difficulties and performance issues regarding bursty traffic of RED, a new technique called controlled delay (CoDel) has been developed. The CoDel is an AQM technique [57] that treats differently low delay queues and queues that continuously buffer packets causing the increased delay. CoDel operates by regularly monitoring the minimum queuing delay in specific intervals and by discarding packets when the minimum queue delay is exceeded. By using queue delay instead of buffer queue occupancy as a queue management metric, CoDel improves network utilization, which further contributes to achieving high throughput and better queue management [58].

Another TCP optimisation method that is used in combination with CC algorithms is the explicit congestion notification (ECN) technique. The ECN is a congestion detection technique based on the setting of the codepoint (mark) in the ECN field of the IP packet header [59]. Based on this codepoint mark annotation, the ECN-capable device can signal on the transport layer regarding the incipient congestion before actual congestion occurs. This allows the CC algorithms to adjust their *cwnd* size accordingly. ECN is generally used in combination with AQM techniques, where ECN marks the IP packet header based on the information obtained from the AQM scheme. Enabling the ECN technique can result in packet loss reduction, queuing delay reduction, and improved throughput in the connection [59]. Instead of dropping packets, an AQM technique may interact with ECN to mark packets and therefore indicate congestion prior to the actual packet loss occurs [60].

Since regular ECN informs the sender only once per RTT about incipient congestion, a more accurate ECN feedback scheme (AccECN) has been proposed. It is based on allowing more than one feedback signal to be transmitted per RTT [61]. Although AccECN was proposed in 2011, it is still an Internet-draft working document expected to be standardised.

### 2.5. Multistream TCP Variants and Alternatives

Although TCP is the dominant transport protocol used today, there are emerging variants and alternatives that can improve overall network performance (Figure 4). The two most relevant protocols are multipath TCP (MP-TCP) and Quick UDP Internet Connection (QUIC). These protocols have been widely used and recently standardized by an Internet Engineering Task Force (IETF). MP-TCP has been introduced as a TCP variant for multipath data communication and QUIC is a UDP-based TCP alternative initially developed for hypertext transfer protocol (HTTP) traffic (Figure 4).

#### 2.5.1. Multipath TCP

Standardized by an IETF in 2020, MP-TCP is an extension to the TCP that handles multiple paths simultaneously for a single data stream [62]. Multipath connection is envisioned to utilize the presence of multiple network interface cards (NICs) commonly found in today’s devices (e.g., smartphones). Hence, MP-TCP can use multiple network interfaces (e.g., 4G, 5G, Wi-Fi, Ethernet, etc.) to create multiple subflows that use multiple paths for a single connection. Using a multipath approach for end-to-end communication can improve utilization of network resources, enhance throughput, and ensure more robust and resilient communication [62].

**Figure 6:**
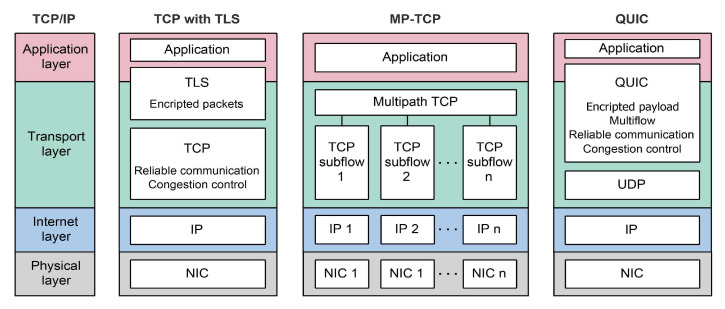
Comparison between TCP with TLS, QUIC and MP-TCP protocol stacks.

In Figure 6, a comparison between the TCP protocol stack and the MP-TCP protocol stack has been presented. It can be seen that MP-TCP divides a single TCP connection into multiple different TCP flows. This dividing requires a CC algorithm that can control the transmission rate for each subflow. To control the data transmission rate for each subflow, MP-TCP CC must satisfy three design goals related to ensuring fair bottleneck bandwidth usage and robustness of the connection [63]. First, multiple subflows of a single multipath connection should perform at least as well as a single path connection would on the best path available. Second, multipath connection should not use more capacity than a single path TCP connection. Finally, an MP-TCP should balance congestion in such a way as to move data towards paths that are less congested. 

Depending on the *cwnd* control method for each subflow, CC mechanisms used with MP-TCP are characterized as uncoupled or coupled. The uncoupled CC mechanism handles each subflow independently allowing each subflow to have its own instance of TCP CC algorithm. On the other hand, the coupled CC mechanism manages *cwnd* in a coupled manner, by considering the characteristics of all other subflows. The initial coupled CC algorithm (Figure 4), called the linked increases algorithm (LIA) defined in Ref. [63], suffers from performance issues. As demonstrated in Ref. [64], LIA transmits a large amount of data over paths that are congested and can have aggressive behavior with respect to a legacy single-path TCP. To address these issues (Figure 4), the authors in Ref. [64] have proposed the opportunistic linked increases algorithm (OLIA). Additionally, authors in Ref. [65] (Figure 4), proposed a balanced linked adaptation algorithm (BALIA). The performance of BALIA is based on balancing responsiveness, TCP friendliness and *cwnd* oscillations. However, these algorithms are based on the legacy TCP Reno CC algorithm and follow the AIMD scheme, and as such, they cannot satisfy previously presented three design goals for operation in a 5G mmWave environment [66]. Since the aforementioned coupled CC algorithms are primarily focused on the increase of the *cwnd,* while neglecting the *cwnd* decreasing mechanism [67] (Figure 4), authors in Ref. [68] proposed a loss-based MP-TCP CC algorithm called Dynamic-LIA (D-LIA). Instead of halving the *cwnd* after packet lost occurrence, the D-LIA decreases the *cwnd* by a dynamically determined factor that depends on the interval between each packet loss [68]. Using this approach, *cwnd* can reach its optimal size much faster than the regular the TCP AIMD mechanism and thus reduce overall network latency and better utilize network resources. Although the D-LIA achieves better overall performance in terms of throughput and fairness, the authors have detected a downside related to increased packet retransmissions [68].

#### 2.5.2. QUIC Protocol

The quick UDP Internet connections (QUIC) protocol is initially developed by Google as an alternative to TCP (Figure 4). It uses user datagram protocol (UDP) at the transport layer (Figure 6). Although standardized in May 2021 by IETF, initial implementation and deployment started in 2012 and QUIC today represents the authenticated and encrypted by default Internet transport protocol with the tendency of eventually replacing TCP and Transport Layer Security (TLS) protocols on the web [69] (Figure 6). QUIC is designed to overcome CC issues of transport and application layer for web-based applications. An upcoming third version of the hypertext transfer protocol (HTTP/3) is designed with QUIC as a built-in transport layer protocol [70] and all major web browsers are starting to support it.

**Figure 7:**
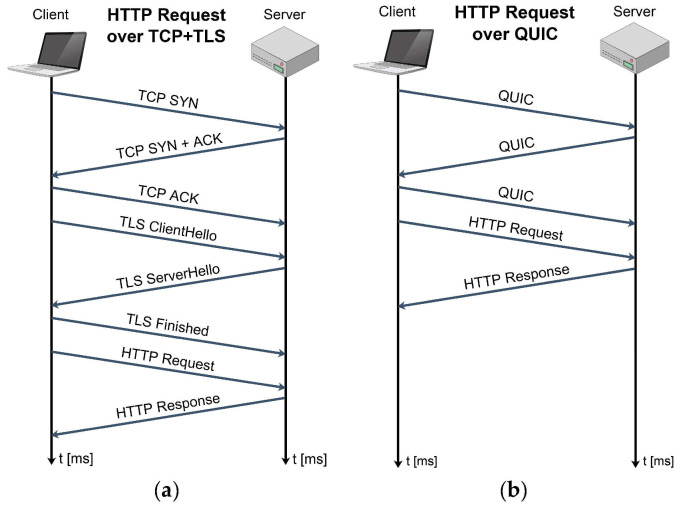
Comparison of connection establishment and message exchange for: (**a**) TCP with TLS, (**b**) QUIC protocol.

According to Figure 6, QUIC includes the TLS layer with its own framing. This ensures permanent authentication and encryption of the connection and makes the initial connection establishment faster. The handshake messages exchange for TCP with TLS and QUIC have been presented in Figure 7. It can be seen that the QUIC handshake only requires one round-trip between client and server to complete, while joint TCP with TLS handshakes requires two round-trips. 

The main advantages of QUIC over TCP include: 0-RTT connection establishment which significantly reduces latency, improves CC with loss detection and minimize head-of-line-blocking delay by supporting the multiplexed operation. Furthermore, QUIC has introduced a pluggable CC interface that provides more flexibility over TCP. Namely, QUIC uses generic CC signaling that can support different CC algorithms, thus allowing flexible algorithm selection [71]. Additionally, QUIC uses monotonically increasing packet numbers to ensure proper packet order. This ensures avoiding retransmission ambiguities and simplified loss detection. It also includes information about the delay between the receipt of a packet and the acknowledgment (ACK) sent for that packet, thus allowing for a more precise RTT estimation. To reduce the packet losses caused by packet bursts, QUIC uses a packet pacing mechanism that evenly spaces packet transmissions over time. It has been shown in Refs. [72,73] that a packet pacing mechanism minimizes the probability of packet losses and supports data stream multiplexing. This eliminates head-of-line-blocking problems what can be especially beneficial over lossy wireless environments (e.g., 4G, 5G networks).

Motivated by the development of the MP-TCP protocol, authors in Ref. [74] have proposed a multipath QUIC (MP-QUIC) protocol (Figure 4). The main capability of MP-QUIC is the possibility to pool resources for a connection that uses multiple paths and to improve resistance to connection failures. This is especially important for today’s multi-homed devices (e.g., smartphones) that need to be able to make an uninterrupted switch between different network interfaces. Multipath extension for QUIC (MP-QUIC) was introduced in 2017 and is described in detail in the IETF Internet draft document, which is currently in the process of standardization [75].

## 3. Related Work on TCP CC Algorithms in 5G mmWave Networks

The performance of the 5G network largely depends on the transport protocol that provides reliable end-to-end communication. As TCP is the most commonly used transport protocol, it has become a necessity for TCP to adapt to new use cases and the requirements of 5G cellular networks. In this section, we have investigated the algorithms used in the most relevant simulation studies for TCP CC in the 5G networks. A comparison of the analysed approaches is presented in Table 2.

In Ref. [76], the authors analysed the TCP CC performance in an end-to-end simulation within different scenarios using the mmWave ns-3 module [77]. In one of the analysed scenarios, the authors simulated the performance of the TCP NewReno algorithm for a mobile user download in a blockage event situation caused by buildings of different sizes. 

The authors evaluated the different situations in this scenario by adjusting the data rates and radio link control (RLC) buffer sizes. The results show that a large buffer induces a severe latency increase due to the bufferbloat problem, especially in a situation where there is a high data rate. In the case of a reduced RLC buffer size, the results have shown that the buffer is too small to accept arriving packets, causing a large number of packet drops. Furthermore, the triggered fast retransmit phase required a very long time period to retransmit the dropped packets, resulting in very poor performance of the TCP NewReno algorithm when a small buffer size was used. The authors conducted another experiment where they observed how the TCP NewReno and CUBIC algorithms reacted to outage events caused by a large building. In a short outage event, both NewReno and CUBIC recovered capacity almost instantaneously due to the lower layer retransmissions. In the long outage event causing RTO expiration, both NewReno and CUBIC showed a slow throughput recovery due to the small *cwnd* size in the slow start phase.

The authors in Ref. [78] studied further issues related to the bufferbloat problem (Table 2). Their analyses are based on simulations using the AQM and proposed dynamic receive window (DRW) mechanisms as potential solutions to the bufferbloat problem (Table 2). The research study compared the performance of the CoDel AQM technique and the cross-layer dynamic receive window adaptation technique over mmWave links. The proposed dynamic receive window mechanism uses a cross-layer design to better estimate the receiver window value, which is dynamically updated based on the optimal BDP [78]. In this simulation study, the authors showed that bufferbloat poses a real problem in the mmWave environment that conventional AQM schemes are unable to address. CoDel AQM in the mmWave environment can reduce latency but it cannot achieve the desired throughput. On the other hand, the authors showed that the proposed cross-layer dynamic receive window scheme can successfully reduce the delay. The delay can be reduced by providing higher channel utilisation without throughput losses [78].

In Ref. [13] the authors conducted a simulation study of TCP in mmWave 5G networks using the four most common CC algorithms: TCP NewReno, HighSpeed TCP, TCP CUBIC and TCP BBR (Table 2). The simulation was performed using the ns-3 mmWave module. For the purpose of the simulation, two challenging scenarios were considered, specifically a high-speed train and a dense urban scenario. The simulations also took into account the edge server and remote server deployment focusing on goodput (application-level throughput) and latency. In the high-speed train scenario, the authors analysed TCP CC’s performance using different RLC buffer sizes and MSSs for the fast-moving UEs served by multiple mmWave NR gNodeB (gNB) base stations. In the dense urban scenario, the authors evaluated TCP CC’s performance where different static UEs are served by a single mmWave gNB. The simulations were performed for the LOS and NLOS conditions using standard MSS and RLC buffer sizes. The authors concluded that moving the server closer to the network’s edge can dramatically improve the latency for all observed scenarios. It was shown that using large buffers can have a positive impact on goodput, but that it can also introduce the bufferbloat problem, causing a latency increase. Conversely, using small buffers provides a lower latency at the expense of a lower goodput induced by the buffer overflow. A trade-off between bandwidth and latency needs to be considered to find the optimal solution. Furthermore, it is shown that the use of a larger MSS size for loss-based CC algorithms can have a remarkable impact on goodput due to the reduced time to reach the full link rate and the rapid recovery after a congestion event. Finally, due to the same buffer size existing for the different channel conditions and network latencies in the dense urban simulation scenario, the authors observed high RTT variability among the CC algorithms.

The authors in Ref. [12] analysed the performance of the TCP CC algorithms in the mmWave network through extensive simulations using the ns-3 mmWave simulation module. Seven different CC algorithms were analysed in a single flow scenario involving different blockage events (Table 2). The authors adjusted the RLC buffer to 7 MB and RTO to 200 ms for the purpose of the simulation. It has been shown that adjusting the RLC and RTO values can have a great impact on all CC algorithms, thus achieving maximum throughput and faster recovery times. The analysis has shown that the Scalable TCP and the CUBIC CC algorithms achieve fast recovery times for short NLOS periods, as opposed to the situation of LOS to extensive NLOS transitions where their performance considerably degrades. On the other hand, the Westwood and the NewReno CC algorithms are shown to be less susceptible to the LOS-NLOS transitions, exhibiting fewer fluctuations in the RTT metrics. The YeAH and BBR algorithms performed similarly, showing robustness in the LOS-NLOS transitions, whereas the BBR exhibited the lowest RTT values among all scenarios (Table 2). The Vegas algorithm has shown a good level of performance regarding RTT at the price of the lowest throughput between the observed CC algorithms. In the second scenario, the authors simulated a single flow with a handover scenario where the mobile user is experiencing short to extensive blockages using the CUBIC and YeAH CC algorithms (Table 2). The simulation showed that, in the case of rapid handover events, the loss-based CUBIC algorithm achieved link capacity quickly whereas the hybrid YeAH performed significantly slower. In the third scenario, the authors analysed the performance of the CUBIC and YeAH algorithms, simulating a scenario of multiple concurrent flows where 10 users were served by 4 BSs (Table 2). Specifically, the short data flows with a background traffic coexistence were simulated with respect of the number of retransmissions and the RLC buffer occupancy [12]. It was shown that the YeAH algorithm outperformed the CUBIC in a number of retransmissions for every usage scenario except one. In the situation where the transmitted data is relatively small, all analysed CC algorithms performed similarly, achieving significantly lower data rates. Regarding the RLC buffer occupancy, in the absence of background transactions with long flows, both CUBIC and YeAH performed similarly. This is as opposed to the situation involving the existence of long flow transactions in the background where CUBIC achieved higher data rates and a higher buffer occupancy. 

New 5G technology supporting mmWave communications will enable a vast number of applications in various industries. The idea of connected vehicles has become more feasible today as new mobile technology emerges. In Ref. [79], the authors compared the performance of conventional TCP CC algorithms in mmWave connected vehicular networks (CVNs) with the proposed real-time wireless TCP (RTW-TCP) CC algorithm (Table 2). It was shown that the high data rates required by CVNs, along with reliable end-to-end communication, represent critical requirements that need to be addressed appropriately. Since the TCP cannot distinguish between channel issues and network congestion, the authors in Ref. [79] detected blockages and beam misalignment as potential issues in mmWave communications in CVNs. The existing CC algorithms, such as Compound TCP, TCP CUBIC, the cross-layer approach to TCP uplink flows (X-TCP) and the proposed RTW-TCP were analysed in various simulation scenarios (Table 2). The proposed TCP mechanism is based on TCP CUBIC with a difference in situation when packet loss occurs. RTW-TCP adjusts its *cwnd* size according to the vehicle mobility and channel quality information (CQI). It reduces *cwnd* size in long blockage situations and maintains *cwnd* size in short blockage situations. The results revealed that RTW-TCP outperforms the other CC algorithms in every simulation scenario in terms of the higher achieved throughput and shorter RTT. The authors concluded that the successful implementation of TCP in mmWave CVNs mandates distinguishing between network congestion and link failures. To achieve the required performance, the algorithm requires adjusting the *cwnd* size accordingly. Finally, the authors noted that the channel characteristics of the mmWave band present a much bigger issue in future CVNs than network congestion. This is the consequence of the fact that congestion will be significantly reduced due to the improved hardware and wider channel bandwidth.

Low latency applications are one of the three main 5G usage scenarios that are expected to be implemented in the mmWave frequency bands. However, as mmWave frequencies are highly susceptible to blockages caused by various objects (e.g., buildings, vehicles, trees) and even the human body, which significantly increases the network delay causing bufferbloat, the use of these frequencies poses a great challenge for low-latency applications [80]. As traditional loss-based CC algorithms are unable to overcome buffer problems, the authors in Ref. [80] conducted an experimental evaluation to examine the behaviour of low-delay CC algorithms in highly variable environments (Table 2). For the purpose of the evaluation purpose, they conducted a CloudLab [82] testbed experiment to explore the behaviour of two low-latency CC algorithms. The TCP BBR and TCP Prague algorithms were compared to the TCP CUBIC algorithm (Table 2). The TCP Prague CC algorithm is part of the low latency, low loss, scalable throughput architecture and it is not a standalone CC algorithm [83]. Four different mmWave link conditions were evaluated in this experiment, including static link, short blockages, long blockages, and mobility with a blockage condition. The results in Ref. [80] confirm that TCP CUBIC is unable to overcome the delay problem in the mmWave environment. Furthermore, in the case of blockages, the ECN-based TCP Prague algorithm shows an increase in delay while still maintaining a lower delay compared to the CUBIC algorithm. It therefore provides good queuing delay management. On the other hand, despite the lack of ECN support, the TCP BBR algorithm maintains low latencies regardless of the link conditions. Moreover, due to the periodical decrease of the *cwnd* size for the purpose of the minimum RTT path estimation, the TCP BBR algorithm may not perform well in applications that require an uninterrupted high-speed service. Regarding the throughput fairness of competing flows, the authors have shown that the TCP Prague algorithm has fairness issues, whereas TCP BBR shows that there is a fair throughput share between the concurrent flows. Finally, the authors noticed that TCP Prague’s deployment, due to the AccECN scheme on both sides of the communication edges, imposes demanding implementation at a large-scale on the public Internet.

The highly variable channel conditions in the mmWave band and the lack of the TCP CC algorithm that can completely alleviate the impact of channel fluctuations on CC performance have led the authors in Ref. [81] to introduce a new Dynamic TCP (D-TCP) CC algorithm (Table 2). D-TCP is an enhanced TCP CC algorithm specifically designed for mmWave 5G networks. D-TCP estimates the available bandwidth to control the *cwnd* considering both the traffic intensity and the varying signal to interference and noise ratio (SINR) fluctuations of the channel. To obtain the SINR information at the transport layer, cross-layer implementation was performed. The authors used an adaptive increase/adaptive decrease (AIAD) paradigm based on the calculated CC factor for adjusting the *cwnd*. In the situation of packet losses, the D-TCP algorithm restored *cwnd* to the previous level with the help of the information related to the SINR variations. Overall, this innovative approach to bandwidth estimation resulted in better network utilisation. 

The authors in Ref. [81] have analysed and compared the performance of the D-TCP with the NewReno, BIC, CUBIC and BBR algorithms using the mmWave ns-3 module (Table 2). The simulations were performed for two different scenarios. They were based on a mobile user served by a single gNB experiencing blockages in small and large building scenarios. In the small building scenario, during the LOS visibility conditions, all CC algorithms performed the same. However, in the situation where the UE experienced NLOS-LOS transitions, there were significant performance differences between the observed CC algorithms. The D-TCP algorithm instantly restored the full bandwidth while TCP BBR and TCP BIC achieved the maximum bandwidth relatively fast. However, TCP CUBIC conducted longer network probing to reach the maximum throughput and TCP NewReno performed the worst regarding the recovery time and achieved throughput. In the large buildings scenario, where the UE is experiencing longer NLOS transmissions, the results showed that the best performance was by D-TCP, which almost instantly reached maximum throughput. This is a consequence of the D-TCP property of adapting to the varying SINR fluctuations in the channel. TCP BBR and TCP BIC performed relatively fast, whereas TCP CUBIC demonstrated a worse result compared to the previous simulation. Finally, TCP NewReno has been shown to have a very poor level of performance.

The presented reach in the related work shows that there is no optimal TCP CC algorithm that can be universally used in 5G mmWave networks. However, different technologies envisioned to be implemented in 5G mmWave networks will impose additional challenges in relation to the realisation of TCP CC. These challenges are discussed in the next section.

## 4. Future Challenges in the Realisation of TCP CC for 5G mmWave Networks

To accommodate the high 5G requirements and complex novel use cases (Figure 1), various new or adapted features and functionalities have been proposed for the practical implementation of 5G networks. These features and functionalities can affect the TCP performance and therefore the user experience. In this section, a brief presentation of the most relevant 5G network features and functionalities is given with a description of their impact on the end-to-end TCP performance (Table 3).

### 4.1. Usage of High Frequencies in the mmWave Spectrum

**Figure 8:**
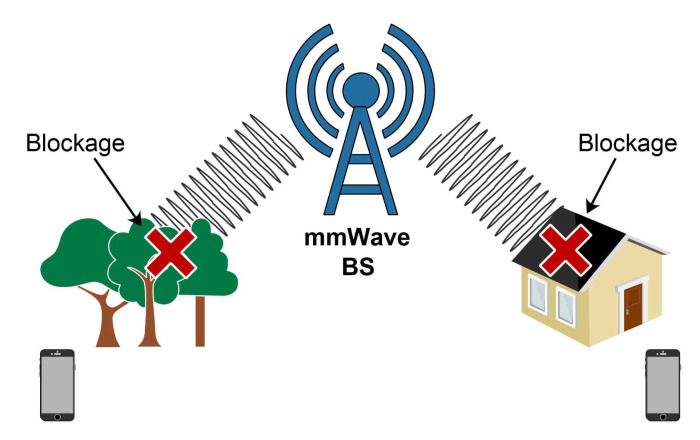
High-frequency millimetre waves experiencing blockages.

The main benefit that the usage of high frequencies in the mmWave spectrum brings is achieving a higher throughput for a massive number of connected devices. Transmission at higher frequencies consequently leads to path loss and blockages since high frequencies cannot penetrate through obstacles (Figure 8). It is expected that due to the frequent LOS-NLOS transitions, the performance of 5 G mmWave networks will be characterised by the occurrence of frequent blockages (Table 3). These blockages cause longer RTTs, higher packet loss probability and RTO expiration, which further degrades the TCP performance over 5G mmWave networks. The negative effects can be more severe for static objects compared to moving objects where there is a higher chance of a faster reconnection between a UE and a gNB. 

Although the use of the mmWave bands ensures that there is a large bandwidth and a high spectral efficiency in the channel, mmWave links suffer from high variability in terms of channel quality due to the signal losses caused by blockages.

To evaluate the performance of the TCP CC in a highly variable mmWave channel, an end-to-end simulation framework was developed in Ref. [76]. This simulation evaluates the performance of the NewReno and CUBIC TCP CC algorithms in a varying 5G mmWave environment. Between the mobile user and the mmWave BS, various obstacles were placed to simulate blockage and outage events. The authors intentionally forced an RTO expiration with two outage events (0.4 s and 1 s long). In the short outage event, *cwnd* never decreased due to the lower layer retransmissions, and both NewReno and CUBIC showed a good level of performance. In the longer outage event, the RTO expired causing TCP CC to enter the slow start phase, reducing *cwnd* to 1 and halving the *ssthresh* size. Although TCP NewReno and TCP CUBIC showed latency reduction, they experienced significant throughput degradation and the slow recovery of the link capacity [76].

Furthermore, the authors in Ref. [12] analysed the performance of multiple CC algorithms using single flow blockage scenarios in a mmWave environment. Several different CC algorithms, classified as loss-based, delay-based, and hybrid, were analysed. The analysis has shown that the longer NLOS periods caused by blockages can affect the performance of the loss-based CUBIC and Scalable TCP algorithms, which repeatedly enter the slow start phase when trying to recover the sending rate. As a consequence of the LOS-NLOS transitions, the results showed the high variability of the RTT metrics. The delay-based Vegas TCP CC algorithm exhibited low RTT values but has the lowest throughput utilisation among the other CC algorithms. The hybrid-based YeAH and BBR TCP CC algorithms displayed a low queuing delay and minimal fluctuations in the RTT metrics, offering low RTT values and throughput comparable to the loss-based CC algorithms. 

The solution required to mitigate the blockage problem in a mmWave band is network densification where the heterogeneous ultra-dense deployment of mmWave BSs will provide more choice of serving BSs for each UE [85] (Table 3). Deploying wireless relays can mitigate the blockage problem as they can effectively restore the communication links. Wireless relays can be based on multi-hop transmissions [86], and the development of TCP CC in such environments represents an important research topic. Furthermore, a solution for high penetration losses can include the use of intelligent reflecting surfaces (IRSs) that can help to bypass the obstacle creating a virtual LOS between the UE and the BS [87].

### 4.2. Horizontal and Vertical Handovers

**Figure 9:**
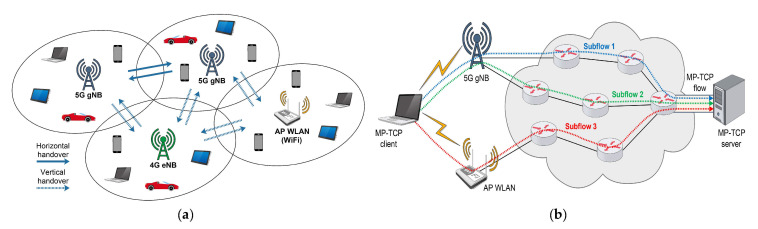
(**a**) Horizontal and vertical handovers, (**b**) Transfer of different MP-TCP subflows.

Considering the upcoming trend characterised by the densification of 5G networks with BSs of different sizes and capacities, it can be expected that a lot of temporary disconnections and connections may happen in the 5G networks between UEs and BSs (Table 3). A horizontal handover is characterised by switching data sessions between different cells in UE situations exiting the specific cell or connecting to a cell that has full capacity. It is likely that this will be a frequent occurrence. Additionally, switching between different wireless technologies (e.g., from 5G to 4G, WLAN, etc.) is known as a vertical handover and this will also be common in the practical implementation of 5G networks (Figure 9a). 

In addition to the aforementioned handovers, in the mmWave 5G networks, the handover can be initiated as a consequence of the high signal penetration loss that can cause blockage events. For example, when a UE connected to a cell experiences a strong degradation of signal level, it must find an adjacent cell to establish a connection. This prevents strong signal degradation and connection termination. The densification of the 5G network with a large number of BSs differing in size and capacity can efficiently alleviate the blockage problem. When a blockage event occurs in networks with an ultra-dense cell deployment, the UE can easily find another cell to maintain the service and connectivity using the handover technique. On the other hand, this network densification in a combination with the blockage occurrence can lead to repetitive handovers, increased handover delays and handover failures. This will cause the UEs to engage in frequent switching between adjacent cells [88] and these handovers can negatively affect the TCP performance by reducing the throughput and increasing delays (Table 3).

The handover impact on TCP performance has been analysed in Ref. [12]. The authors performed a simulation using two scenarios, a single flow with a handover and multiple concurrent flows, where the TCP CUBIC and YeAH TCP CC algorithms were compared. Since the handover in cellular networks refers to a scenario where an ongoing data session is transferred from one BS to another to ensure user connectivity while moving, in mmWave networks, besides user movement, the handover can be caused by a blockage. The single flow simulation showed that short-term blockages initiate the frequent switching of UEs between BSs, which causes degradation in the throughput. Two of the observed CC algorithms showed different recovery times in the case of rapid handover events, where CUBIC achieved an appropriate link capacity quickly while YeAH performed significantly slower. In the simulation scenario of multiple concurrent flows where multiple users are served by multiple BSs, the authors observed a negative handover impact due to the occurring blockages. Namely, when a UE moves through the environment and consequently switches between two BSs, it causes the throughput degradation of the UE that is already connected to the BS to which the moving user is connecting.

Network condition changes due to frequent blockages caused by repetitive horizontal or vertical handovers pose a real challenge in the implementation of 5G communications [89]. To address these challenges, different handover management schemes as presented in Refs. [88,90,91,92] have been considered. Moreover, the possible implementation of MP-TCP with devices containing multiple NICs and TCP flows over each NIC could represent a solution to the negative impact of frequent handovers on CC in 5G networks (Figure 9b). In the case of multiple NICs and corresponding TCP CC algorithms, the blockage of flow on one NIC will not affect flow on other NIC(s). This can contribute to uninterrupted transmission and ensured CC among communicating ends. However, devices working with multiple active NICs in parallel, will not be energy efficient. This particularly can affect sensors and battery-supplied devices. Hence, the search for the most appropriate management scheme in 5G mmWave networks is still an open research topic. Possible solutions will be dedicated to the customisation of the management schemes devised for the different services for which a specific variant of TCP is used. 

### 4.3. Implementation of the 5G Core Network

**Figure 10:**
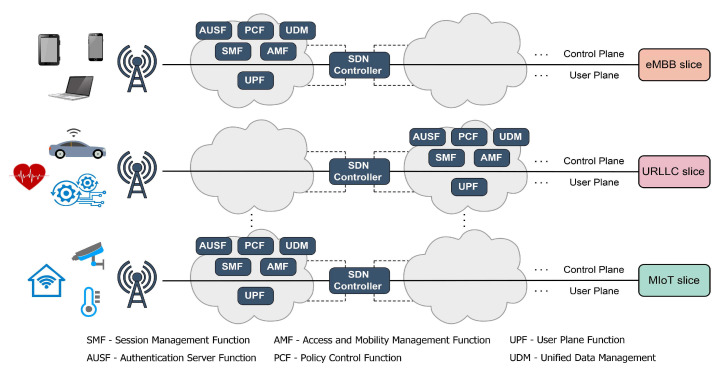
Control and user plane separation supported through network slicing.

The 5G Core Network (5GC) specified in Ref. [93] is an evolution of the 4G LTE Core or Evolved Packet Core (EPC) architecture [94]. In contrast to EPC which includes different function modules as dedicated hardware, the 5GC architecture is designed as a service-based architecture (SBA) provided by a set of network functions (NFs). Moreover, 5GC introduces the mechanism that includes the separation of the user plane from the control plane functions, in addition to support for network slicing (Figure 10) in different 5G usage scenarios (Figure 1) [95]. The deployment and management of different NFs in the 5G heterogeneous networks with various service requirements present a significant challenge to the practical realisation of 5G networks. To ensure the parallel and isolated functionality of the different services and network slices and to guarantee the separation of the user and control plane, the implementation of software-defined networking (SDN) and network function virtualisation (NFV) techniques are considered to be possible solutions [96,97].

However, a huge number of simultaneously supported services, the possible existence of a large number of different network slices and the separation of the data and user plane traffic can affect the performance of the TCP (Table 3). The modular design of NFs can be leveraged to deploy different CC algorithms for different services, thus ensuring optimal network performance. High-speed TCPs can be used for delay-sensitive services or services requiring high data rates. The implementation of a 5GC network ensures the realisation of a standalone 5G network where the full potential of the service-based approach can be exploited by offering services based on the system requirements. Each network slice can be optimised for the required use case using an adequate TCP CC algorithm, ultimately leading to a better end-to-end user experience (Table 3). The impact of new features supported by the 5GC network on TCP performance represents an important future research topic and needs to be analysed in detail.

Since the user plane function (UPF) is responsible for the user plane operations (i.e., packet routing/switching and gateway selection), finding a high-performance UPF for fast packet processing can significantly decrease the latency [98]. This can indirectly improve the overall TCP performance. Finding optimal user plane management techniques for 5G, as presented in Ref. [99], could be another solution to further improve TCP performance.

Due to the increasing popularity of QUIC as a TCP alternative, the author in Ref. [100] has conducted a testbed experiment of QUIC performance in a 5G core network for satisfying different quality of service (QoS) criteria. The author has shown that QUIC is more resilient than TCP in a 5G core network under poor network conditions (high packet loss rate, high latency, and low bandwidth) and under optimal network conditions as well. Additionally, due to the faster connection establishment compared to TCP, the author has confirmed that QUIC can achieve significantly better performance in the 5G core network. Hence, the examination of the possible implementation of QUIC in 5G core networks represents an open research field. 

### 4.4. Usage of Beamforming

**Figure 11:**
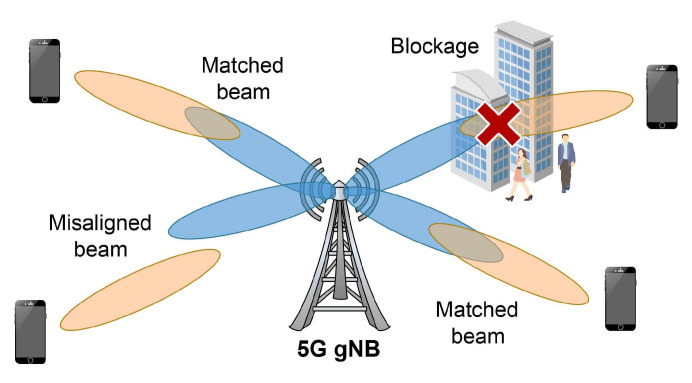
Beamforming scenarios in 5G mobile networks.

For the transmission of signals from BS to UE, the use of massive antenna arrays in combination with beamforming techniques can alleviate the mmWave signal propagation issues such as high path losses (Table 3). Beamforming improves the coverage and signal quality by focusing the powerful signals toward a particular device (Figure 11). The precise alignment of directional beams in a mmWave 5G network requires advanced beam management algorithms. This can be ensured by the use of effective control layer procedures [101], such as initial access [102] and beam tracking [103]. However, a misalignment or mismatch between the transmitter beam and receiver beam can lead to a low receiving gain, reduced throughput or connection loss. Furthermore, forming the directional beams between gNB and the UE can compensate for a high path loss. On the other hand, it can cause a blockage problem due to the high beam directivity (Figure 11) [104]. Misalignment problems can severely degrade the TCP performance (Table 3), especially in a high mobility scenario where the beam mismatch occurs more frequently causing intense SNR fluctuations. These fluctuations can cause intermittent interruptions that can vary based on the size of obstacles and the speed of the UEs (from short to long disconnections). Although both disconnections can affect the performance of the TCP, the effects of the long ones are stronger [105]. This is due to the high probability of triggering the RTO, which leads to a congestion window initialisation and the dramatic slowing of the sending rate. 

To address the impacts of beam misalignment and blockage problems on TCP performance in the mmWave frequency bands, the authors in Ref. [104] have proposed a deep-learning-based TCP (DL-TCP) algorithm. The proposed algorithm is compared against the existing NewReno, CUBIC and BBR TCP CC algorithms in a disaster 5G mmWave network where frequent blockages and beam mismatches are expected. To address the beam misalignment problem, the beam sweeping technique was used. The simulation study showed that the DL-TCP algorithm brings in improvements in performance when compared to other CC algorithms in 5G disaster environments. This is a consequence of the deep learning approach used by the DL-TCP algorithm. Such an approach can predict the link disconnection time when a packet loss occurs and can adjust the *cwnd* size accordingly.

Due to the large amounts of bandwidth and high spectral efficiency, the 5G mmWave frequencies can enable multi-gigabit data rates. However, transmission at such high frequencies imposes high signal propagation losses and the usage of highly directional beams is necessary to compensate for these losses. To effectively use one of the beamforming techniques, it is necessary to overcome these issues. The solution to the beamforming issues in a challenging mmWave environment can be found in the efficient beam management techniques [101] combined with a robust beam tracking strategyand the implementation of a customised TCP CC algorithm [106]. The implementation of these combined approaches and strategies currently represent an open research topic. 

### 4.5. Implementation of Edge Computing 

One of the three main 5G use cases is the possibility of enabling URLLC services (e.g., remote healthcare, intelligent transportation and real-time services). The URLLC requires ultra-low latencies for critical applications characterised by ultra-high reliability and availability (Figure 1). In 5G networks operating in the mmWave bands, latency can be seriously degraded due to blockages or beam misalignment. Large delays in the 5G network strongly impair TCP performance (Table 3). It is crucial to reduce the end-to-end transmission latency in the mmWave 5G networks, especially when the loss-based TCP algorithms are implemented. Using TCP optimisation techniques such as the AQM can contribute to the mitigation of the RTT delay to some extent. However, for the URLLC usage scenarios, the network infrastructure needs to be adapted to meet these high requirements. 

**Figure 12:**
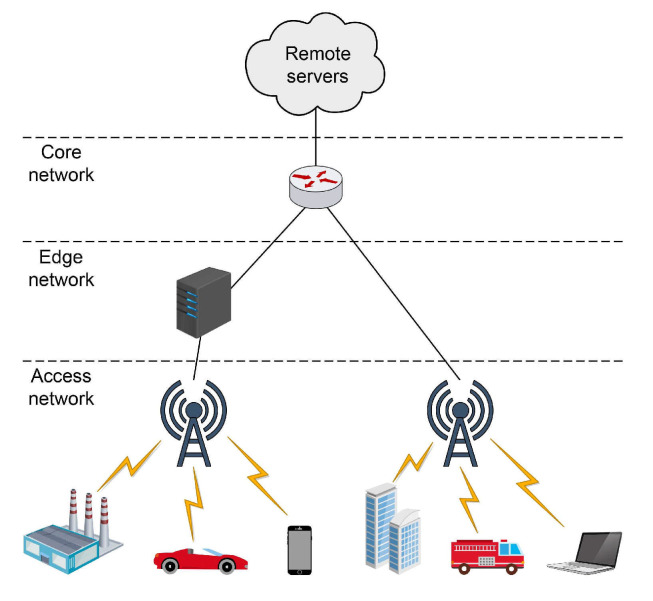
Edge computing model.

One of the emerging technologies that can address these issues is edge computing. Edge computing is developed to overcome traditional cloud computing problems (such as high latency) by bringing cloud capabilities closer to the users (Figure 12) [107]. Since the RTT value depends on the sum of the three main factors of communication, computational and propagation delay, it can be expected that the full implementation of edge computing can greatly improve the overall TCP performance. By providing storage and computational capabilities near to the location of the users, edge computing efficiently addresses the propagation delay by shortening the distance between the communication ends. Besides the possible improvement of the TCP performance, the implementation of edge computing can consequently reduce the communication delay by offering higher data rates [107].

The impact on TCP performance using remote servers and edge server deployment has been analysed in Ref. [13] using UT high-speed movements in dense urban scenarios. For both scenarios, the authors compared several loss-based CC algorithms and the hybrid BBR algorithm. The comparison was performed for a setup involving a remotely deployed server having long RTT and a mobile edge computing (MEC) deployment [108]. The server was located in the proximity of the gNB, which ensures low latency. The results show that in a high-speed scenario, edge server deployment greatly reduces the RTT for all of the observed CC algorithms. In terms of the achieved goodput, edge server scenarios have a positive impact in most situations due to the presence of loss-based algorithms. However, for the BBR algorithm, the results showed a negligible improvement. 

The authors have shown that, in the dense urban scenario, only the TCP BBR in the edge server deployment can satisfy the typical 5G requirements (goodput larger than 100 Mbps and a latency lower than 10 ms) operating under stable channel conditions. As presented, the usage of edge computing technology can greatly improve the data rates and decrease latency, especially for loss-based algorithms. The reason for this is due to the shorter control loop resulting from the shorter distance between the UEs and the server, which leads to a faster reaction to congestion. However, further investigations are needed in order to deduce how TCP CC in different 5G use cases can benefit from the implementation of edge computing technologies.

### 4.6. Implementation of Buffering for Radio Link Control 

**Figure 13:**
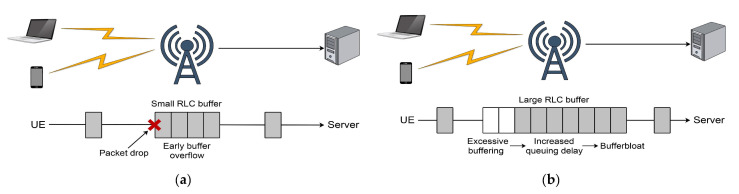
Different buffer size implementations: (**a**) packet drops due to a small RLC buffer, (**b**) bufferbloat problem due to a large RLC buffer.

To prevent packet drops caused by high transmission link occupancy, the RLC layer performs a temporal buffering of the packets (Figure 13). The size of the RLC buffer can have a significant impact on the TCP performance (Table 3). Deploying large buffers prevents buffer overflow and reduces the chance of packet drops (Figure 13b). On the other hand, large buffers can dramatically increase latency, which can be a consequence of the long queuing delays. Such delays can cause bufferbloat problems in the mmWave 5G networks (Figure 13b). Reducing the buffer size can mitigate the bufferbloat problem. However, reducing the buffer size can also have a negative impact on the TCP performance (Figure 13a). More specifically, using small buffers can greatly affect the performance of the TCP CC algorithms, especially those that are loss-based, due to the high packet losses in a buffer overflow situation (Table 3). Additionally, high link variations of the mmWave channels can result in frequent NLOS communication periods. These periods can cause a faster buffer overflow what consequently leads to frequent packet drops and decreased throughput.

The described issues related to the selection of the optimal buffer size in the RLC of mmWave 5G networks motivates investigations which analyse the impact of buffer size on the TCP performance. More specifically, the authors in Ref. [12] analysed the impact of different RLC buffer sizes on the TCP CC performance. The analysis showed that the frequent packet losses caused by small buffer sizes prevented loss-based algorithms such as CUBIC from reaching the congestion avoidance phase. Furthermore, the authors in Ref. [10] showed that the best results in terms of the TCP CC can be achieved when the optimal RLC buffer size is used in combination with a reduced RTO value.

Ref. [13] analysed the impact of RLC buffer size on goodput and latency. A performance comparison among the different loss-based algorithms, such as TCP NewReno, HighSpeed TCP, TCP CUBIC, and hybrid TCP BBR, was undertaken. The authors showed that large buffers can offer a higher goodput. This is the consequence of the higher robustness of the mmWave channel quality variations and the lower possibility of buffer overflow. Nevertheless, it is shown that using large buffers significantly increases latency. This is due to the large buffer occupancy, which can further lead to bufferbloat problems. It is also shown that deploying small buffers decreases the latency at the expense of goodput.

Additionally, the authors analysed the usage of large buffers in combination with the CoDel AQM technique. In comparison to the scenario lacking AQM technique, the obtained results show a decrease in goodput in relation to the loss-based CC algorithms. However, the decreased goodput was still larger than that obtained by the small buffer size. It is further shown that applying AQM for loss-based CC algorithms contributes to the significant latency reduction, which is comparable to the levels of latency reduction in the case of small buffer deployment. The hybrid TCP BBR algorithm in a remote server scenario using AQM showed a large decrease in latency and the same goodput as when no AQM was applied. 

The authors showed that the best goodput was the hybrid TCP BBR algorithm in combination with a large buffer size. However, the obtained goodput was still under the maximum achievable rate. A downside was that the hybrid TCP BBR with a large buffer size had the highest latency compared to the other CC algorithms. 

According to the presented analyses, it can be seen that an inappropriate RLC buffer size can cause buffer overflow or the bufferbloat problem. This can lead to an increased packet latency or packet drops respectively. Determining the optimal size of the RLC buffer and using different AQM techniques, such as CoDel and Flow Queue CoDel [84], can ensure a trade-off between throughput and latency. Finding solutions that will ensure an optimal buffer size in the RLC layer of mmWave 5G networks represents an open research topic. Further research attempts are needed to address this issue. 

### 4.7. Constantly Transmitted Signals

**Figure 14:**
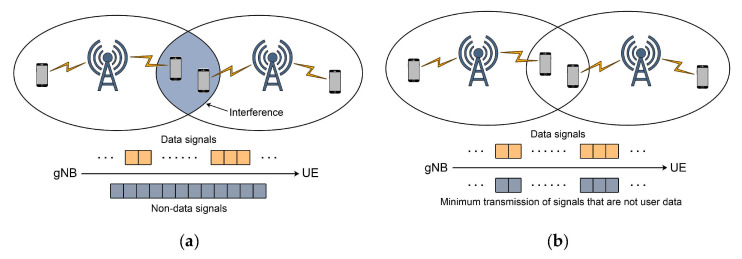
Activity of the radio network based on (**a**) always-on signals, (**b**) ultra-lean design.

Besides the user traffic signals, in mobile networks, BSs transmit signals at regular intervals used for different purposes such as BS detection, synchronisation, system information broadcasts, channel estimations etc. [109] (Table 3). The transmission of these signals is done regardless of the user traffic (Figure 14a). Although these signals will not consume a significant percentage of the overall channel usage, 5G will be based on ultra-dense cell allocation. These “always-on” signals can pose a problem in emerging 5G heterogeneous networks (Figure 14). Due to the constant nature of the transmissions, these signals can create interference between the adjacent cells, which further reduces the throughput (Table 3). The constant transmission of signals has a negative impact on the overall energy consumption of the cellular network [110,111]. The increased traffic in combination with the always-on signals causing increased interference can affect the TCP performance in terms of the increased number of congestion events (Table 3). This leads to a degraded throughput and raises issues related to the fairness of the distribution of the TCP flows for different users.

The main advancement in 5G mobile networks dedicated to the mitigation of negative effects of constantly transmitted signals is the implementation of an ultra-lean design in the network (Table 3). The aim of the network’s ultra-lean design is to minimise the transmission of non-data signals (Figure 14b). This means that non-data signals should be transmitted only when necessary. Such an approach enables longer sleep periods for the BSs, which consequently improves the BSs energy efficiency [110]. Furthermore, the implementation of an ultra-lean design in the 5G networks will reduce the level of interference, which consequently reduces the congestion events. This can lead to improved TCP functionality (Figure 14b). However, deeper investigations are needed regarding the impact of the ultra-lean design on TCP performance. Since in the ultra-lean design non-data signals will be reduced or switched off until they are needed, TCP algorithms must be appropriately adopted to these working principles. This imposes the development of new approaches on the TCP flow control.

## 5. Machine Learning for Improving CC

Today, 5G cellular networks are emerging with a large number of new use cases and applications. Conventional TCP CC algorithms that have been implemented in the last few decades are rule-based and their performance in next generation networks is often suboptimal. Conventional CC algorithms base their decisions on pre-defined criteria such as packet loss or delay and they lack the ability to learn and adapt their behaviour in complex and changing environments such as contemporary 5G cellular networks. Ref. [112] detected several constraints related to conventional CC algorithms. The main constraint is the inability to adapt to new networks where, for example, the algorithms that are designed for wired links will not perform efficiently in wireless networks. Another important constraint is an inability to learn from the previous experiences based on past information. This reduces the possibility of achieving the full potential of modern networks and results in sub-optimal performance. It is expected that using machine learning (ML) techniques will effectively overcome the limitations of the conventional TCP CC algorithms.

As opposed to conventional CC algorithms that use predefined parameters as a measure of performance effectiveness, the ML approach exploits learning techniques in order to adapt to network dynamics (Table 4). The ML CC solutions can be categorised into one of three main approaches of offline learning, online learning and deep reinforcement learning (DRL). The offline learning technique involves the definition of the network and traffic models for specific algorithms. The ML process and algorithm generation are then performed offline. To determine the optimal mappings, they are performed before the actual network implementation. The main drawback of this approach is that the emulated network and traffic model in which the algorithm is optimised may differ from the actual live network, therefore such an approach provides sub-optimal results [113]. As shown in Table 4, some of the ML CC algorithms that use offline learning are the Remy [114] and Indigo [115] ML algorithms. 

Furthermore, the online learning approach does not use fixed mappings and pre-defined actions. Instead, this approach uses the ML techniques in a live environment that adapt to network conditions on the fly. This approach provides a better level of performance when changing the network environment where the network parameters cannot be accurately predicted. If the network model is accurately predicted and corresponds to the actual network environment in which the algorithm is implemented, then the offline models outperform the online models [113]. The performance-oriented congestion control (PCC) [116] and the PCC Vivace [117] are examples of ML CC algorithms that use online learning techniques (Table 4). 

The DRL is an ML technique that uses reinforcement learning (RL) and deep learning methods (Table 4). It is a decision-making paradigm modelled as a Markov decision process (MDP) [121]. In the framework of this approach, the DRL agent, through combining RL and deep neural networks (DNN), learns from interacting with the environment. In terms of the CC, adjusting the *cwnd* size or increasing the sending rate can be viewed as the actions of the DRL agent in the decision-making process. The strategies of taking actions under the actual network environment are based on the policy that maximises the expected cumulative reward. The reward metric can be observed as either the throughput or network delay that is optimised for a particular system state by the actions of the DRL agent using trained DNN [112]. Some of the CC algorithms that use DRL techniques include Aurora [118], Eagle [119] and Orca [120] (Table 4).

As the ML is evolving, its application in the CC field becomes an area of particular research interest and this will become the main concept exploited in the research dedicated to the improvement of CC in 5G networks [122,123,124]. To the best of our knowledge, the aforementioned ML CC algorithms have not yet been evaluated using end-to-end simulations in a mmWave environment. In future research, it is expected that different ML CC algorithms will be extensively tested in various experimental 5G mmWave scenarios. Researches have yet to see how this cutting-edge technology will perform, especially in comparison with the existing CC algorithms in mmWave 5G networks.

## 6. Research Challenges and Future Directions

It has been shown that there is no unique TCP CC solution that can satisfy all use cases and applications, especially in a highly variable environment such as 5G mmWave networks. Developing an efficient transport layer protocol that is able to effectively utilise the mmWave bandwidth and overcome the issues in 5G network CC, such as blockages, misalignment, and handover, presents a great challenge. Future challenges are manifested in the development of an optimal solution for a particular situation. For example, in emerging usage scenarios where one device requires a high bandwidth priority and the other requires ultra-low latencies, the use of different TCP CC algorithms is expected. Scenarios in which multiple different TCP flows are controlled using different CC algorithms and compete for their fair share of the link capacity can pose a serious challenge in terms of practical realisation. More specifically, according to the presented research and the conducted simulations, the problem of fairness for different TCP flows has not yet been successfully solved. To prevent fairness issues, critical applications, such as autonomous driving or telesurgery, where ultra-high reliability and ultra-low latency are expected, should be controlled by only one TCP CC algorithm in an isolated environment.

Another challenge is to find a solution that will utilise the full channel bandwidth and minimise latency at the same time. Queuing delays as a consequence of large bottlenecks at the buffer level must be addressed as their impact can seriously deteriorate network performance. One of the solutions that is promising is the cross-layer approach where the transport layer of the Open System Interconnection (OSI) model can use the information obtained from the different layers to adjust the *cwnd* size accordingly.

Regardless of the method used for congestion detection, from the analysis of the presented works, we have addressed the three main challenges to TCP CC when operating in the mmWave mobile network:

- Latency vs. throughput trade-off challenge: There are various solutions to achieve low latencies. However, many of them are at the expense of bandwidth. It will be a challenge to find a solution that will achieve the best compromise for the chosen scenario.
- Queuing delay and bufferbloat problem: The problem of excess packet buffering, which creates very large queuing delays known as bufferbloat, was detected in many research studies. Nevertheless, it is a research area that still needs to be further explored in the mmWave band.
- Fairness problem: This occurs in non-isolated environments such as the public Internet where multiple competing TCP flows use different CC algorithms. Fairness emerges as one of the main problems and it is an area where further research is needed to find the optimal solution.

Although CUBIC is the dominant CC algorithm for the broad internet traffic today, the BBR algorithm increased its share in terms of the practical implementation and it can be expected to become the dominant algorithm in the future. As the BBR algorithm is slowly replacing the CUBIC algorithm, further research regarding their mutual interaction is needed to ensure the stability of the Internet. Despite the fact that BBR achieves good performance in terms of maximising the throughput and minimising latency, in highly variable mmWave environments with a massive number of connected devices, achieving an optimal network performance will be challenging. It can be concluded that in the near future, the use of BBR will be sufficient for eMBB usage scenarios, especially if it becomes the dominant algorithm. However, further research is needed to implement TCP CC in the upcoming Internet of Everything (IoE) concept characterised by a large number of installed sensors. This will use 5G mobile networks as an infrastructural backbone. 

Academic and industry researchers are constantly making efforts to improve traditional rule-based algorithms that use predefined heuristics to address new requirements. On the other hand, ML TCP CC is in its early stages and it remains to be seen whether the future will be consistent with the traditional CC paradigm or if the future of TCP CC lies in intelligent ML algorithms. The deployment of the ML in TCP CC is still in its infancy and it is too early to expect any significant application at a higher level. However, with the advent of 6G networks, ML will need to be considered as the dominant direction in the field of network CC. There are possible directions in the implementation of the ML for CC. The first one is based on the development of entirely new ML-based algorithms that will completely replace the existing ones. The second is based on the integration of some of the ML properties into the existing algorithms and maintaining its backward compatibility with rule-based algorithms. The future will show which of these directions will dominate. 

## 7. Conclusions

The 5G mobile networks must accommodate high demands and support various use cases. Besides sub-6 GHz frequency bands, the new mmWave spectrum is considered in the 5G mobile communications due to the high spectral efficiency, high spatial reuse, low latencies, and multi-gigabit data rates. However, the implementation of the 5G network in sub-6 GHz and mmWave frequency bands brings new challenges in the realisation of TCP CC.

In this paper, a comprehensive survey of TCP algorithms used for CC is presented. An overview of surveyed algorithms includes single-flow, multi-flow TCP CC algorithms, and alterative algorithms that have been envisioned as the future replacement for TCP. TCP and the concept of CC have been presented through an overview of the relevant related works that are primarily focused on the current cognitions concerning the challenges in the improvement of CC functionality in the 5G networks. The CC implementation challenges in the 5G networks can be caused by blockage problems, beam misalignment, frequent handovers, inadequate buffer sizes, interference due to the constant transmission of non-data signals, and changes in the data flow due to the usage of edge computing. These challenges can degrade the TCP CC performance and, in this work, each challenge has been explained. The current research attempts for alleviating the issues related to every challenge have been elaborated on. Due to the challenges and limitations that conventional CC algorithms involve, it is realistic to expect further advancements of the TCP CC algorithms in the years to come. 

These advancements are also expected in the implementation of the ML techniques, which been overviewed in terms of improving CC in the 5G networks. Therefore, the most relevant smart ML-based CC algorithms with the ability to learn and adapt to future complex networks are overviewed. Finally, a discussion related to the main challenges that must be addressed for the efficient implementation of CC algorithms in the upcoming 5G mobile networks has been presented. The discussion emphasises the potential directions in the development of ML-based algorithms as the most promising candidates for the implementation of CC in complex 5G and future 6G networks.

## Figures and Tables

**Table 1:** Overview of the most relevant single-flow TCP CC algorithms.

CC Algorithm	Introduction Year	Type	Features
Tahoe [22]	1988	Loss-based	Slow start, congestion avoidance and a fast retransmit mechanism.
Reno [27]	1990	Loss-based	Introduced fast recovery mechanism.
NewReno [29]	1999	Loss-based	Fast recovery modification to allow multiple retransmissions.
SACK [30]	1996	Loss-based	Selective ACK option.
Westwood [31]	2001	Loss-based	Introduced a faster recovery mechanism that controls the sending rate according to the available bandwidth estimation.
HighSpeed [32]	2003	Loss-based	Introduced a modified TCP response function to allow for a faster *cwnd* increase and a faster recovery time in situations with a high *cwnd* size.
Scalable TCP [33]	2003	Loss-based	After each received ACK during the RTT, algorithm increases the *cwnd* size in proportion with the defined constants. After the packet loss decreases *cwnd* by a smaller factor, the standard CC is exploited.
BIC [34]	2004	Loss-based	Uses the binary search increase and additive increase techniques to determine the *cwnd* size.
CUBIC [35]	2008	Loss-based	Uses the cubic function for *cwnd* control characterised by a steady state and a maximal probing behaviour.
Vegas [36]	1994	Delay-based	Modification of TCP Reno that predicts network congestion before an actual loss of segments occurs. Uses a fine-grained RTT estimation and has a very efficient segment retransmission schedule.
Vegas+ [37]	2000	Delay-based	Modification of TCP Vegas that introduced an aggressive mode to overcome fairness issues when competing with TCP Reno.
New Vegas [38]	2005	Delay-based	Implemented three server-side modifications of TCP Vegas to overcome performance issues in a high latency environment.
Vegas-A [39]	2005	Delay-based	Implemented modified congestion avoidance mechanism to address fairness, rerouting and bias against high bandwidth connections issues of TCP Vegas in wired and satellite networks.
Vegas-V [40]	2012	Delay-based	Modification of TCP Vegas-A that addresses fairness and aggression issues when competing with TCP Vegas, TCP Vegas-A and TCP Reno flows.
FAST [41]	2004	Delay-based	Designed for high-speed long-latency networks. Adjusts the *cwnd* according to the feedback information of the average RTT and average queuing delay. Uses scaling parameters to effectively utilise the network capacity.
Compound [42]	2006	Hybrid	Based on the loss-based slow start phase. During the congestion avoidance phase, it uses a combination of two components, a standard loss-based and a new scalable delay-based component.
YeAH [43]	2007	Hybrid	Performs a dynamic exchange between a slow mode during the congestion avoidance phase and a fast mode during the fast recovery phase.
BBRv1 [44]	2016	Hybrid	Builds an explicit model of the network using the estimated RTT and the estimated available bottleneck bandwidth in order to prevent congestion.
BBRv2 [45]	2018	Hybrid	Uses ECN signals, improved fairness with CUBIC and lower packet loss rates for the optimisation of the TCP CC performance.

**Table 2:** Review of the research articles on CC algorithms in 5G networks.

Simulation Scenarios	Evaluated TCP CC Algorithms	Main Algorithm Drawbacks	Summary
UE experiencing LOS to NLOS transitions and outage events between mmWave BS in small buildings and large building scenarios [76].	NewReno	Large buffer: bufferbloat. Small buffer: early buffer overflow. Long outage: throughput degradation and slow throughput recovery.	Evaluated loss-based CC algorithms showed slow reaching of full throughput, large data rate drops, increased latency and slow throughput recovery.
	CUBIC	Long outage: throughput degradation and slow throughput recovery.	
UE experiencing blockages from other humans and from buildings [78].	Cubic with AQM CoDel	Human blockage: packet drops, slow throughput recovery. Building blockage: multiple packet drops resulting in near-zero throughput.	AQM CoDel does not mitigate the bufferbloat problem and DRW showed a much higher throughput and negligible oscillation in the delays.
	CUBIC with DRW	Low delay and much higher throughput in both scenarios.	
High-speed train scenario with different buffer sizes and a dense urban scenario, using remote server and edge server deployment [13].	NewReno	Remote server: lowest goodput.	Latency is greatly reduced for all observed CC algorithms using edge server deployment. Applying the AQM scheme with loss-based CC algorithms can reduce the latency in large buffer deployments.
	CUBIC	Edge server: lowest goodput.	
	HighSpeed	Big buffer: high latency and goodput.	
	BBR	Big buffer: high latency and goodput. Small buffer: low latency with weak goodput reduction.	
UE experiencing blockages between mmWave BS in extensive blockages, medium blockages and multiple short blockages scenarios. Handover scenario between three BSs and a mobile user experiencing multiple short to extensive blockages. Dense small cell deployment with various obstacles in a situation of multiple BSs serving multiple UEs when short flows and background traffic coexist [12].	NewReno	Blockage events: Slow full throughput reach after multiple losses and slow network probing in the congestion avoidance phase.	Blockage events greatly impact latency for loss-based CUBIC and Scalable TCP. Delay-based Vegas showed the lowest throughput with minimal latency variability. Hybrid CC algorithms showed minimal performance variations. Loss-based CUBIC showed high-performance variations in longer NLOS periods as opposed to hybrid YeAH which showed minimal throughput variations and required fewer transmissions, but achieved less throughput compared to CUBIC.
	CUBIC	Blockage events: High RTT variability in LOS-NLOS transitions. Handover: fast throughput recovery from the slow start. Multiple flows: high number of retransmissions and high buffer occupancy, high throughput.	
	Scalable TCP	Blockage events: High RTT variability in LOS-NLOS transitions.	
	Vegas	Blockage events: Low throughput with minimal RTT variability.	
	Westwood	Blockage events: Slow network probing in congestion avoidance phase.	
	YeAH	Blockage events: Low RTT and minimal performance variability. Handover: slow throughput recovery from slow mode. Multiple flows: low number of retransmissions and high robustness.	
	BBR	Blockage events: Low RTT in all scenarios and minimal performance variability.	
Multiple BSs serving multiple vehicles moving at random speed in the mmWave CVNs environment using two different mobility models in rural and urban areas [79].	CUBIC	High *cwnd* size variability.	Due to the high channel fluctuations caused by mobility in CVNs, the RTW-TCP outperformed the existing CC algorithms as they cannot distinguish between congestion and link failures.
	Compound	High average RTT, lowest aggregate throughput and high *cwnd* size variability.	
	X-TCP	Low average RTT and high *cwnd* size variability.	
	RTW-TCP	Low throughput reduction due to mobility, low RTT and *cwnd* continued to increase despite blockages.	
Multiple UEs communicating with single mmWave access point under static link, short blockages, long blockages, and mobility and blockages scenarios [80].	CUBIC	Long queuing delay and good fairness.	CUBIC showed a dramatic increase in the delays in NLOS conditions. BBR is not suitable for uninterrupted high-speed applications and Prague has fairness issues.
	BBR	Low queuing delays and good fairness. Periodically reducing sending rate.	
	Prague with DualQ, AQM and AccECN	Lowest queuing delay and poor fairness.	
Single gNB serving mobile users in a small building and large buildings scenario [81].	NewReno	Lowest performance.	D-TCP using cross-layer implementation to obtain SINR information showed the best performance among the evaluated CC algorithms.
	BIC	Relatively fast achieves full throughput.	
	CUBIC	Long network probing.	
	BBR	Relatively fast achieves full throughput.	
	D-TCP	The best performance and almost instantly achieves full throughput.	

**Table 3:** Challenges and possible solutions to the realisation of TCP CC with respect to 5G main mmWave network functions.

mmWave 5G Network Function	Reasons for Implementation in 5G Networks	Realisation Challenges in the 5G Network	Realisation Challenge Concerning TCP Performance	A Possible Solution to the Challenge
Frequent horizontal and vertical handovers	Reduces outage occurrence and blockage occurrence. Improves network energy efficiency, UE signal strength or BS capacity.	A lot of temporary disconnections and connections may happen in the network.	Frequent handovers can confuse the TCP when scaling its congestion window size. This reduces the capability of TCP to ensure low packet drops.	Ensuring appropriate throughput levels and TCP CC through the development of optimal handover algorithms. Implementation of devices with multi NICs and CC with multipath TCP protocols.
Usage of high frequencies in the mmWave spectrum	Transmission at higher frequencies ensures higher throughputs.	A blockage occurs since high frequencies (in mmWave spectrum) cannot pass through obstacles.	Blockages can cause the frequent triggering of TCP RTOs, longer RTTs and increase the probability of packet losses. When compared with moving UEs, these negative effects can be more evident for static UEs, since moving UEs have a faster chance of reconnection with gNB or UE.	Extending the LOS areas of the network. Putting in wireless relays in order to keep the LOS communication and optimal allocation and densification of heterogeneous network elements composed of BSs differing in size and capacity. Using intelligent reflective surfaces.
Usage of beamforming for transmission of signals from BS to UEs	Improves the coverage and signal quality by focusing the powerful signals toward a particular device.	Mismatch between the beams of the transmitter and receiver that reduce or completely eliminate the possibility of connection. Mismatched beams can cause long or short interruptions that can impact the performance of TCP.	Prevent TCP from establishing reliable end-to-end connections. High end-to-end throughput degradation in the case of NLOS communication since SNR at the location of UE cannot reach the expected values. The longer interruptions have a stronger impact on TCP performance due to the higher probability of triggering the RTOs, which further initialises the congestion window and slows the sending rate.	Development of advanced beamforming algorithms and beam tracking concepts. Implementing beam sweeping techniques, which tend to establish communication pairs after beam mismatch occurs.
Implementation of a 5G core network to support: service-based architecture, network slicing, SDN/NFV concepts	Ensures data transmission between different parts of radio access networks through a core 5G network. Ensures the realisation of a stand-alone 5G network.	Ensuring the parallel and isolated functionality of different services. Enabling appropriate separation among the different network slices. Implementation of user and data planes in separate SDN/NFVs.	TCP end-to-end congestion and flow control issues due to: the large number of simultaneously supported services, the existence of a huge number of different network slices, the separation of data and user plane traffic.	For services with high data rates, high-speed TCP CC algorithms can be used. For delay-sensitive services, the appropriate TCP CC algorithms can be deployed. Separation of the control- and user-plane with the optimal selection of distinct TCPs for each one. Implementation of QUIC protocol for CC of multiplexed web streams in core networks.
Implementation of buffering for radio link control	Enables the compensation of packet losses for higher-layer protocols.	An optimal algorithm for the selection of the buffer size. The optimal selection of the buffer location.	Implementing large buffers can cause long TCP queues. Long waiting by the packets in buffers leads to bufferbloat problems and higher latencies. Implementing small buffers decreases latency but in the case of high channel variations, an increase in dropped packets can occur. Such an increase in the number of packet losses due to reduced buffer size strongly affects loss-based TCPs.	Development of new techniques that will ensure a trade-off between performance and latency. Implementation of adjusted AQM techniques such as CoDel [57] and Flow Queue CoDel [84].
Constantly transmitted signals	Signals that enable base station detection, system information broadcasts, channel estimation, etc.	Constantly transmitted signals are independent of the UEs traffic. Such transmission consumes a part of the network capacity and negatively impacts on the energy consumption of the network devices (BSs)	Constantly transmitted signals contribute to the increase of redundant traffic and network interference. This affects the TCP performance in terms of CC and the fair distribution of data flows among the users.	Implementation of 5G networks based on the ultra-lean design based on smart signalling exchange. The ultra-lean design can reduce traffic and congestion events, and improve the TCP functionality.
Using edge computing with the support of network slicing	Reduction of the network latency through the optimised allocation of computing resources.	Large delays in the 5G network negatively impact the TCP’s functionality, especially those based on loss-based TCP protocols.	Implementing separate TCP algorithms in each slice to ensure optimal CC. Part of the applications deployed in the user-plane between the core and access network.	Solutions concerning the allocation of the servers close to UEs using approaches based on content delivery network (CDN). Development of novel TCP CC algorithms customised to the needs of a specific network slice.

**Table 4:** Machine learning CC algorithms.

Algorithm	Learning Method	Main Algorithm Characteristics
Remy [114]	Offline	Based on network and traffic models. Uses pre-specified objectives for CC and the lookup table for maximising the expected value of the objective function.
Indigo [115]	Offline	Congestion control oracles are generated that map the algorithm state to correct actions using an emulated network model. The training data is generated by applying an imitation learning algorithm that uses CC oracles.
PCC [116]	Online	Learning is based on live experimental evidence and the utility function which describes an objective. Uses multiple micro-experiments to make a rate control decision and gradient ascent-based online learning algorithm.
PCC Vivace [117]	Online	A variant of the PCC algorithm that uses a learning-theory informed framework. In addition to the bandwidth and loss rates, the proposed framework includes RTT gradients for utility function derivations.
Aurora [118]	DRL	Uses a small fully connected neural network model with changes in the sending rates such as agent actions. Computes statistics vectors based on latency gradient, latency ratio and the sending ratio. The algorithm is based on a fixed-length history of the statistic vectors representing its states. The algorithm gives rewards in terms of throughput improvements while penalising latency and packet losses using a linear reward function. The ML approach is based on the Proximal Policy Optimisation (PPO) algorithm in order to train DRL agents.
Eagle [119]	DRL	Model training is based on a long-short term memory (LSTM) neural network. The cross-entropy method is used to train a DRL agent. Uses a summary of the past four observation states and different reward functions for different cases. The actions of the ML agent regulate the discrete changes in the sending rate and the *cwnd* size.
Orca [120]	DRL	Uses DRL coarse-grain control and classic TCP CC schemes for fine-grain control. This ML approach is based on the recurrent neural network model. Exploits the twin delayed deep deterministic policy gradient (TD3) as the training algorithm. The reward function is calculated using packet delivery rate, delay and loss, which averages the values to compose the state space.

## Abbreviations

The following abbreviations are used in this manuscript:

3G	3rd Generation Mobile Network
3GPP	3rd Generation Partnership Project
3WHS	Three-Way Handshake
4G	4th Generation Mobile Network
5G	5th Generation Mobile Network
5GC	5G Core Network
6G	6th Generation Mobile Network
AccECN	Accurate Explicit Congestion Notification
ACK	Acknowledgment
AIAD	Adaptive Increase Adaptive Decrease
AIMD AMF AP	Additive Increase Multiplicative Decrease Access and Mobility Management Function Access Point
AQM AUSF	Active Queue Management Authentication Server Function
BALIA	Balanced Linked Adaptation Algorithm
BBR	Bottleneck Bandwidth and Round-Trip Propagation Time
BDP	Bandwidth-Delay Product
BIC	Binary Increase Congestion Control
BS	Base Station
BtlBw	Bottleneck Bandwidth
CC	Congestion Control
CoDel	Controlled Delay
CQI	Channel Quality Information
*cwnd*	Congestion Window
D-LIA	Dynamic Linked Increases Algorithm
DL-TCP	Deep-Learning-Based Transmission Control Protocol
DNN	Deep Neural Network
DRL	Deep Reinforcement Learning
DRW	Dynamic Receive Window
D-TCP	Dynamic Transmission Control Protocol
ECN	Explicit Congestion Notification
eMBB	Enhanced Mobile Broadband
eNB	Evolved Node B
EPC	Evolved Packet Core
gNB	Next Generation Node B
HARQ	Hybrid Automatic Repeat Request
IETF	Internet Engineering Task Force
IoE	Internet of Everything
IoT	Internet of Things
IP	Internet Protocol
IRS	Intelligent Reflecting Surface
ITU-R	International Telecommunications Union–Radiocommunications Sector
IW	Initial Window
LIA	Linked Increases Algorithm
LOS	Line-of-Sight
M2M	Machine to Machine
MAC	Media Access Control
MDP	Markov Decision Process
MEC MIoT	Mobile Edge Computing Massive Internet of Things
ML	Machine Learning
mMIMO	Massive Multiple-Input and Multiple-Output
mMTC	Massive Machine-Type Communication
mmWave	Millimeter-Wave
MP-TCP	Multipath TCP
MP-QUIC	Multipath QUIC
ms	millisecond
MSS	Maximum Segment Size
NF	Network Function
NFV	Network Function Virtualization
NIC	Network Interface Card
NLOS	Non-Line-of-Sight
OLIA	Opportunistic Linked Increases Algorithm
OSI	Open System Interconnection
PCC PCF	Performance-Oriented Congestion Control Policy Control Function
QoS	Quality of Service
QUIC	Quick User Datagram Protocol Internet Connection
RL	Reinforcement Learning
RLC	Radio Link Control
RTO	Retransmission Time-Out
RtProp	Round Trip Propagation Time
RTT	Round-Trip-Time
RTW-TCP	Real-Time Wireless Transmission Control Protocol
rwnd	Receiver Window
SA	Standalone
SACK	Selective Acknowledgment
SBA	Service-Based Architecture
SINR SMF	Signal to Interference and Noise Ratio Session Management Function
*ssthresh*	Slow-Start Threshold
SYN	Synchronize
TCP	Transmission Control Protocol
TFO	Transmission Control Protocol Fast Open
TLS	Transport Layer Security
TMRG UDM	Transport Modeling Research Group Unified Data Management
UDP	User Datagram Protocol
UE	User Equipment
URLLC WiFi	Ultra-Reliable and Low Latency Communication Wireless Fidelity
WLAN	Wireless Local Area Network
YEAH	Yet Another Highspeed Transmission Control Protocol
